# Single-cell transcriptomic profile unveils distinct immune ecosystems in alcohol- and nonalcohol-associated hepatocellular carcinoma

**DOI:** 10.3389/fimmu.2026.1882068

**Published:** 2026-08-18

**Authors:** Huan Huang, Haotian Chen, Xutao Xu, Shuo Pan, Yuheng Cheng, Junliang Tang, Qiang Wang, Junyue Tao, Lijuan Huang, Renbiao Chen, Chengtao Jin, Hongxia Zhang, Yanfei Fang, Kan Wang, Qian Cao, Zhenzhen Wen

**Affiliations:** 1 Department of Gastroenterology, Sir Run Run Shaw Hospital, Zhejiang University School of Medicine, Hangzhou, China; 2 CAS Key Laboratory of Tissue Microenvironment and Tumor, Shanghai Institute of Nutrition and Health, Chinese Academy of Sciences, Shanghai, China; 3 Department of Gastroenterology, Cixi People’s Hospital, Wenzhou Medical University, Ningbo, China; 4 Huashan Hospital, Fudan University, Shanghai, China; 5 Nanjing Medical University, Nanjing, China; 6 General Surgery, Cancer Center, Department of Hepatobiliary & Pancreatic Surgery and Minimally Invasive Surgery, Zhejiang Provincial People’s Hospital (Affiliated People’s Hospital), Hangzhou Medical College, Hangzhou, China; 7 Department of Gastroenterology, the First People’s Hospital of Wuyi, Jinhua, China; 8 Department of General Practice, Sir Run Run Shaw Hospital, Zhejiang University School of Medicine, Hangzhou, China; 9 Department of Radiology, Sir Run Run Shaw Hospital, Zhejiang University School of Medicine, Hangzhou, China; 10 Department of Pharmacy, Sir Run Run Shaw Hospital, Zhejiang University School of Medicine, Hangzhou, China

**Keywords:** alcohol-associated hepatocellular carcinoma, immunosuppression, single-cell RNA sequencing, Treg, tumor microenvironment

## Abstract

Alcohol-associated hepatocellular carcinoma (A-HCC) shows more aggressive progression and has a poorer prognosis than nonalcohol-associated HCC (NA-HCC), but the underlying tumor microenvironment (TME) heterogeneity remains poorly characterized. Here, we performed single-cell RNA sequencing (scRNA-seq) on tumor and adjacent normal tissues from two A-HCC patients, constructing the first single-cell transcriptomic profile of human A-HCC. This dataset was integrated with public NA-HCC scRNA-seq data to compare cellular composition, functional states, and intercellular communication. Both HCC subtypes displayed immunosuppressive TME features, which were significantly more pronounced in A-HCC. Specifically, A-HCC tumors showed greater enrichment of regulatory T cells (Tregs) with enhanced suppressive function, anti-inflammatory macrophage polarization, and more severe CD8^+^ T and NK cell depletion and dysfunction with reduced metabolic activity. Malignant hepatocytes in A-HCC exhibited increased copy-number variation, epithelial-mesenchymal transition (EMT), stemness, proliferation, and drug resistance associated gene signature scores. Cell-cell communication analysis further suggested A-HCC-specific upregulation of immunosuppressive signaling pathways (LTA/LTB, TGF-β, LGALS9-HAVCR2) that reinforce this pro-tumorigenic ecosystem. A Treg-derived gene signature from A-HCC was associated with worse overall survival in independent TCGA cohorts. This first comprehensive single-cell comparison uncovers a coordinated immunosuppressive and malignant ecosystem in A-HCC that may be distinct from NA-HCC, providing a mechanistic framework to understand its aggressive clinical behavior and to identify potential etiology-targeted therapeutic strategies. Despite the limited sample size, this study provides the first single-cell resource for A-HCC and lays the foundation for future large-scale validation.

## Introduction

Liver cancer ranks as the sixth most common malignancy and the fourth leading cause of cancer-related mortality worldwide (1). Hepatocellular carcinoma (HCC) is the predominant histological subtype, accounting for approximately 90% of all primary liver cancers (2). Alcohol is a major etiological driver, contributing to roughly 30% of global HCC cases and 19% of HCC-related deaths. Moreover, the age-standardized mortality rate for alcohol-associated HCC (A-HCC) has increased by 0.53% annually in recent years (3). Chronic heavy alcohol consumption leads to a spectrum of liver disease, ranging from steatosis and steatohepatitis to cirrhosis, and eventually to HCC. Despite alcohol being a well-established risk factor for HCC development and progression, a substantial gap remains between basic research and clinical application. This gap largely stems from the lack of robust preclinical models for A-HCC, particularly when compared with the well-established models for nonalcohol-associated HCC (NA-HCC) caused by etiologies such as viral hepatitis.

Clinically, A-HCC is more aggressive than NA-HCC. It’s often diagnosed at an advanced stage typically with underlying cirrhosis, and exhibits higher mortality rates (4, 5). Part of the reason for this poor prognosis is that patients with alcohol-associated liver disease (ALD) are not adequately screened and have poor adherence to follow-up. Patients with non-alcoholic fatty liver disease (NAFLD) face similar screening challenges (6). However, when examining outcomes, ALD patients have a slightly higher HCC incidence, worse survival (7), and higher recurrence rates (8) than NAFLD patients. These differences may arise from distinct pathological backgrounds. Most A-HCCs develop in cirrhotic livers, whereas 41.7%-75.0% of NA-HCCs occur in non-cirrhotic livers (9). Furthermore, although ALD and NAFLD share some histological features such as steatosis, ballooning, and lobular inflammation (10–12), ALD tends to exhibit more severe pathological changes (13, 14). Adding to this complexity, steatosis is also observed in hepatitis virus-related HCC, but there it’s mainly located in the periportal area rather than around the central vein, suggesting that different etiologies lead to distinct metabolic perturbations (15, 16). Despite all these clinical and pathological clues, the molecular mechanisms underlying the prognostic disparity between A-HCC and NA-HCC remain poorly understood. Therefore, a high-resolution dissection of the drivers of these two HCC subtypes is urgently needed.

The tumor microenvironment (TME) of HCC is a complex and dynamic system. Immune cells, in particular, play a key role in disease progression and treatment response (17). Unlike many other solid tumors, HCC progression is heavily shaped by the composition and functional state of infiltrating immune cells. Thus, profiling the immune landscape at high resolution is critical for improving immunotherapies and discovering new biomarkers. Recent advances in single-cell RNA sequencing (scRNA-seq) have made this possible in HCC. Multiple studies have mapped the immune and malignant cell microenvironment of NA-HCC, revealing extensive immunosuppressive features and transcriptionally distinct subpopulations (18–23). Despite alcohol being a well-established global risk factor for HCC, A-HCC remains poorly characterized compared with NA-HCC (24). To data, only one study used scRNA-seq in a mouse model of alcoholic liver injury and identified alcohol-induced transcriptional alterations in hepatocytes, endothelial cells, and Kupffer cells (25). Whether these findings translate to human A-HCC, and how the A-HCC TME differs from NA-HCC at single-cell level, remain unknown.

Here, we built the first single-cell transcriptomic profile of A-HCC, using tumor and adjacent normal tissues from two patients. We then integrated this dataset with public single-cell transcriptomic data from NA-HCC to construct a high-resolution landscape of cellular heterogeneity, including immune cells, non-immune cells, and intercellular communication networks. In tumor tissue of A-HCC, we observed a profoundly immunosuppressive microenvironment relative to adjacent normal tissue, characterized by depletion of CD8^+^ T cells and natural killer (NK) cells, accompanied by enrichment of regulatory T cells (Tregs) and macrophages. This immunosuppressive state was further reinforced by functional shifts in key immune subsets, including that Tregs displayed stronger immunosuppressive signature, macrophages leaned toward an anti-inflammatory phenotype, and CD8^+^ T cells appeared dysfunctional and exhausted in A-HCC. Meanwhile, A-HCC hepatocytes acquired more malignant traits, including increased stemness, proliferation, and drug-resistance signature scores. Cell-cell communication analysis suggested upregulation of immunosuppressive signaling pathways (LTA/LAB, TGF-β, LGALS9-HAVCR2) that reinforce this pro-tumorigenic ecosystem. Collectively, our study points to a distinct immunosuppressive architecture coordinated across multiple cell lineages in A-HCC and identifies etiology-specific cellular programs that may underlie its aggressive behavior. These findings advance our understanding of the diversity of the HCC tumor ecosystem and provide a conceptual framework for designing etiology-tailored immunotherapies, an increasingly important direction in cancer immunology.

## Materials and methods

### Human tissue processing

Two male patients with a history of long-term alcohol consumption (≥40 g ethanol/day for more than 5 years) were enrolled from Sir Run Run Shaw Hospital, Zhejiang University School of Medicine. Both patients were clinically diagnosed with alcohol-associated HCC (A-HCC) after excluding viral hepatitis (HBV/HCV negativity), nonalcoholic fatty liver disease, autoimmune liver disease, and other parasitic and inherited metabolic disorders. Diagnosis was based on clinical symptoms, laboratory tests, imaging examinations, and, when necessary, liver biopsy. None of the patients received any anti-tumor treatment prior to surgery. Patients with mixed etiologies (e.g., alcohol plus viral hepatitis) were excluded.

Fresh cancerous and adjacent non-cancerous tissue samples were collected from both patients, immediately rinsed in ice-cold phosphate-buffered saline (PBS), and subsequently preserved in MACS^®^ Tissue Storage Solution (Miltenyi Biotec, 130-100-008).

### Single cell isolation from surgical specimens

The samples were minced into small pieces and digested with collagenase II (Worthington) and DNase I (Worthington), then passed through a 70µm cell strainer. After centrifugation, the supernatant was removed, and the cell pellet was resuspended in erythrocyte lysis buffer (Miltenyi Biotec) to remove red blood cells. The cells were washed with PBS containing 0.04% bovine serum albumin (BSA), resuspended, and filtered through a 35µm strainer. The resulting single-cell suspension was stained with acridine orange/propidium iodide (AO/PI), and cell viability was assessed using a Countstar fluorescence cell analyzer.

### Single cell sorting, partitioning, and library preparation

Single-cell RNA-seq libraries were generated using the 10× Genomics Single Cell Preparation System and Chromium Single Cell 3’ Reagent Kit v3.1 (10× Genomics, Pleasanton, CA, USA). Cells were concentrated to approximately 1,000 cells/uL and loaded into each channel to generate gel bead-in-emulsions (GEMs), which serve as single-cell microreaction systems for reverse transcription. Following reverse transcription, GEMs were broken, and barcoded cDNAs were purified and amplified. The final libraries were quantified using a High-Sensitivity DNA chip (Agilent) on a Bioanalyzer 2200 and a Qubit High Sensitivity DNA assay (Thermo Fisher Scientific). Sequencing was performed on an Illumina sequencer (Illumina, San Diego, CA, USA) with a paired-end 150bp read length.

### scRNA-seq data processing and quality control

All samples were processed using Cell Ranger pipeline (v5.0.1) (10×Genomics, v.3.1). Raw sequencing data were demultiplexed, and FASTQ files were generated with cellranger mkfastq. Adapter sequences were trimmed and low-quality reads were removed using fastp (v0.23.4) with default parameters to obtain clean data (26). The cleaned reads were aligned to the human genome (GRCh38, Ensemble version 100) using cellranger count to generate feature-barcode matrices.

The UMI count matrices were converted into Seurat object using the R package Seurat (v4.4.0) (27). Genes detected in fewer than three cells were excluded, and only genes overlapping with the NA-HCC scRNA-seq dataset were retained. Cells with fewer than 200 or more than 4,000 detected genes were removed. Cells with more than 30% of UMIs derived from mitochondrial genes were also discarded to eliminate dead or dying cells. Doublets were identified and removed using DoubletFinder (v2.0.3) (28). After filtering, 20,852 high-quality single cells from A-HCC samples were retained for downstream analysis.

### NA-HCC data acquisition and preparation

The scRNA-seq count matrices of NA-HCC were downloaded from the GSE149614 dataset (29), which contains 21 samples, including 10 primary tumor tissue, 8 corresponding peritumor liver tissues, 1 metastatic lymph node sample, and 2 portal vein tumor thrombus samples. Since our study focused on comparing tumor and normal tissues, we selected corresponding tumor and peritumor samples. Sample HCC07T (only 510 cells) and its matched peritumor sample (HCC07N) were excluded. After filtering, a total of 48,003 single-cell transcriptomes from seven tumor samples and their matched peritumoral tissues were included.

Gene symbols in the expression matrix were updated using the complete HGNC dataset to ensure consistency. Only genes overlapping with the A-HCC scRNA-seq dataset (17,285 genes) were retained for further analysis.

### Data integration, clustering, and cell type annotation

The filtered gene expression matrices were normalized. Highly variable genes (top 2000 features) were identified using FindVariableFeatures with default parameters. To integrate samples across the tissues and patients, the top 2000 features were used with SelectIntegrationFeatures. Canonical correlation analysis (CCA) was performed for batch correction using FindIntegrationAnchors, followed by IntegrateData to remove batch effects and merge datasets. To systematically evaluate integration performance, we compared five methods, principal component analysis (PCA), harmony, mutual nearest neighbors (MNN), single-cell variational inference (scVI), and canonical correlation analysis (CCA) using the Local Inverse Simpson’s Index (LISI), a metric that quantifies local mixing of cells from different batches (30). CCA achieved the highest LISI score and was therefore selected as the primary integration method (**Supplementary Figure 1A**). The integrated data were scaled, and principal component analysis (PCA) was performed using the top 2,000 variable genes (dimensions = 25). Cell nearest neighbor networks were constructed with FindNeighbors, and clustering was performed using the Louvain algorithm with FindClusters (resolution = 0.4). For visualization, uniform manifold approximation and projection (UMAP) was applied using RunUMAP.

Cell clusters were annotated by comparison with a reference dataset using SingleR (v2.0.0) (31), combined with known canonical markers. Major immune cell types were identified, including Tregs, NK/NKT and CD8^+^ T cells (*CD3E*, *CD3G*, *CD8A*, *CD8B*, *CD4*, *FOXP3*, *KLRF1*, *KLRD1*, *NCAM1*), B cells and Plasma (*CD79A*, *MS4A1*, *CD19*, *IGHD*, *IGHG1*, *TNFRSF17*), and myeloid cells (*LYZ*, *CST3*). Major non-immune cell types included hepatocyte (*ALB*, *TTR*, *APOA2*), endothelial cells (*PECAM1*, *CDH5*), and hepatic stellate cells (HSCs; *COL1A1*, *ACTA2*, *PDGFRB*).

### Differentially expressed genes in specified cell types

To assess functional differences of specific cell types between A-HCC and NA-HCC, differentially expressed genes (DEGs) were identified using the FindMarkers function in Seurat with parameters logfc.threshold = 0.01 and min.pct = 0.01. Genes with fold change ≥ 1.2 and adjusted p-value < 0.01 were considered statistically significant.

### Functional enrichment analysis

Genes were ranked by log_2_ fold change values obtained from differential expression analysis (FindMarkers in Seurat). Gene set enrichment analysis (GSEA) for Kyoto Encyclopedia of Genes and Genomes (KEGG) pathways was performed using the gseKEGG function in the R package clusterProfiler (v4.6.2) with parameters pvalueCutoff = 0.05, minGSSize = 10, and maxGSSize = 500. Results were visualized with the R packages enrichplot (v1.18.4) and ggplot2.

### scRNA-seq signature score calculation

Gene signature scores for specific cell subpopulations were calculated using the AddModuleScore function in Seurat. Pro-inflammatory and anti-inflammatory signature genes for macrophages were based on published research (32). Glycolysis and tricarboxylic acid (TCA) cycle signature score for CD8^+^ Te, NK and NKT cells were based on published research (33). The following gene sets were evaluated for hepatocytes based on published research, including liver malignant signature (34), epithelial-mesenchymal transition (EMT) gene signature (35), stemness signature (36), cell cycle-related genes (KEGG pathway hsa04110) and platinum drug resistance genes (KEGG pathway hsa01524).

### CNV estimation

We inferred large-scale chromosomal copy number variations (CNV) from single-cell transcriptome profiles using the R package infercnv (v1.14.2) (37). inferCNV uses a sliding window approach to compare relative gene expression intensities across cells, with a predefined set of reference cells serving as a diploid baseline. In our analysis, T cells and myeloid cells were used as the diploid reference set, as these immune cells are considered to have normal copy numbers. The analysis was run with a cutoff of 0.1. After initial CNV calculations, we applied denoising to the final heatmap using the denoise function, which removes random noise from the CNV signal and improves the resolution of true copy number changes. CNV scores were calculated for each cell by averaging the final denoised CNV values across all genomic windows. Cells with a CNV score above the 95th percentile of the reference cells were considered malignant. These scores were then used to compare malignancy levels between A-HCC and NA-HCC hepatocytes.

### Cell-cell communication analysis

To investigate ligand-receptor mediated signaling changes between cell types, we used CellChat (v1.6.1) to analyze and compare the cell-cell communication networks between two HCC subtypes (38). CellChat infers and analyzes communication networks from single-cell transcriptomic data by integrating gene expression with a curated database of literature-supported ligand-receptor interactions. The human CellChat database (CellChatDB.human) was used as the reference for ligand-receptor pairs. For each HCC subtype, we extracted the corresponding tumor sample from the Seurat object and created separate CellChat objects using the createCellChat function. Overexpressed ligands and receptors in each cell group were identified using identifyOverExpressedGenes and identifyOverExpressedInteractions with default settings. Communication probabilities between cell groups were calculated using computeCommunProb. To ensure reliability, we filtered out communications involving cell groups with fewer than 10 cells using filterCommunication(min.cells = 10). Communication probabilities were then aggregated at the signaling pathway level using computeCommunProbPathway, and the overall networks were summarized with aggregateNet.

To compare communication patterns between A-HCC and NA-HCC, the two CellChat objects were merged using mergeCellChat. Differential signaling analysis was performed to identify context-specific signaling pathways and up-regulated and down-regulated ligand-receptor pairs between the two conditions. Information flow for each signaling pathway, defined as the sum of communication probabilities among all pairs of cell groups, was calculated and compared using rankNet. Significant ligand-receptor pairs were defined as those with p < 0.05 and log_2_ fold change > 0.1 between A-HCC and NA-HCC. For each ligand or receptor gene, expression levels across groups and cell types were visualized using the VlnPlot function of Seurat.

### Survival analysis

Public gene expression and survival data for HCC patients were obtained from TCGA datasets (Firehose Legacy and PanCancer Atlas) via the cBioPortal public datahub (https://www.cbioportal.org/). Among the 10 hepatocellular carcinoma datasets available on the platform, these two were the only ones containing both RNA-seq transcriptomic data and complete overall survival information. Samples with missing values and overall survival less than one month were excluded (Firehose Legacy: n = 342; PanCancer Atlas: n= 338).

To identify a Treg-related prognostic signature, we first selected genes that were upregulated in A-HCC tumor Tregs compared to NA-HCC tumor Tregs, with the following criteria: adjusted p-value < 0.01, fold change > 1.5, and the percentage of expressed cells at least 15% higher in A-HCC than NA-HCC. Univariate Cox regression analysis was then performed on these genes, and those significantly associated with overall survival (p<0.01) were defined as the A-HCC Treg prognostic signature. To evaluate whether the signature provides independent prognostic value beyond basic clinical covariates, we performed multivariate Cox regression analyses adjusting for age and sex using the coxph function in the survival R package.

To evaluate the combined prognostic effect of these signature genes, gene set variation analysis (GSVA) was applied to calculate a signature score with mx.diff using GSVA R package (v.1.46.0). Risk scores were predicted by predict function of survival R package for the Cox model based on GSVA enrichment scores. Patients were then grouped into high- and low-risk score groups based on the median risk score.

All survival analyses and hazard ratio (HR) calculations were performed using the R package survival (v3.5-7). Survival curves were visualized with survminer (v0.4.9), and forest plots for univariate Cox regression results were generated using the forestplot R package (v3.1.3).

### Statistical analysis

Comparisons of gene expression or gene signature score between two groups of cells were performed using unpaired two-tailed Wilcoxon rank-sum tests. All statistical analyses and data visualization were conducted using R software (version 4.2.2).

## Results

### Cell landscape and shared immunosuppression in A-HCC and NA-HCC

To investigate the cell heterogeneity of A-HCC and compare it with NA-HCC at single-cell resolution, we performed scRNA-seq on four samples from two A-HCC patients, including two primary tumors (Tumor) and two matched adjacent non-tumor liver tissues (Normal) (detailed clinical information in **Supplementary Table 1**; expanded clinical metadata in **Supplementary Table 2**). To enable comparative analysis, we integrated the newly generated dataset with a publicly available NA-HCC scRNA-seq dataset (GSE149614). After quality control, the final integrated cohort comprised 18 samples from nine patients, including two A-HCC and seven NA-HCC cases, with each contributing one tumor and one adjacent tissue sample. A-HCC cohort includes only two patients; therefore, our findings should be interpreted as exploratory.

**Figure 1:**
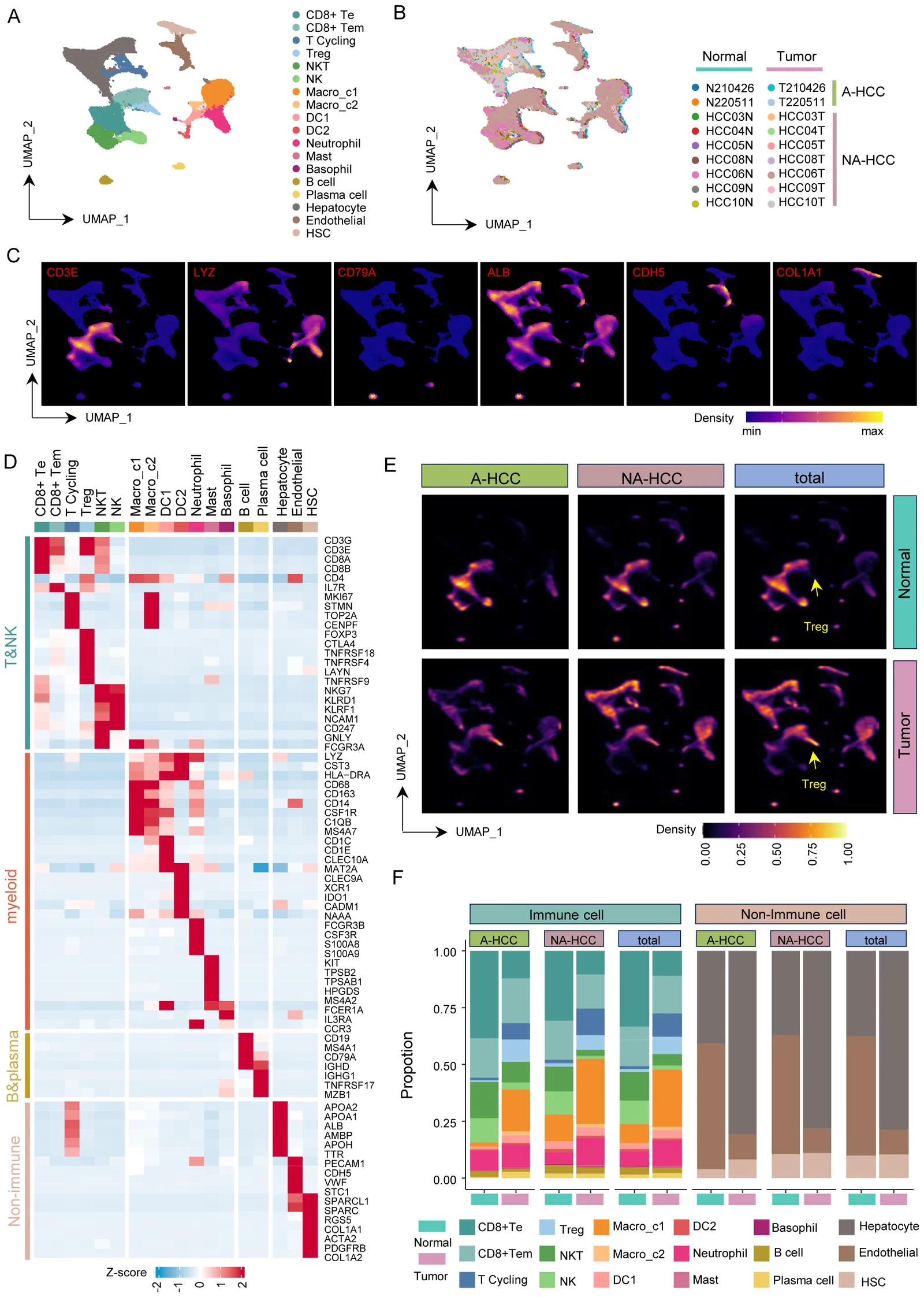
scRNA-seq profiling of the A-HCC and NA-HCC tumor environments. **(A)** UMAP plot of all cells, colored by annotated cell types. **(B)** UMAP plot colored by samples. Tumor: tumor tissues; Normal: adjacent normal tissues. The same applies to subsequent figure legends. **(C)** UMAP plots showing expression density of marker genes for the major cell types (indicated). **(D)** Heatmap of marker gene expression across cell types. The top bar corresponds to the cluster annotation in **(A)**. **(E)** UMAP plots showing cell distribution density across two HCC subtypes (A-HCC vs. NA-HCC) and tissue types (Normal vs. Tumor) as indicated. **(F)** Bar plots showing proportions of immune and nonimmune cell type in each group, colored by cell type.

After quality control and doublet removal, we integrated the datasets using five integration methods (PCA, Harmony, MNN, scVI and CCA) and evaluate using LISI, which identified CCA as the optimal approach (**Supplementary Figure 1A**). We obtained 68,855 high-quality cells for downstream analysis. The median gene detection rate was 1,702 genes per cell, with a median of 6,829 unique molecular identifiers (UMIs) per cell. The cell composition was balanced between tumor (49.8%) and adjacent normal tissues (50.2%). Unsupervised clustering and uniform manifold approximation and projection (UMAP) dimensionality reduction identified 18 distinct cell clusters (**Figure 1A**). All cells expressed housekeeping genes (*ACTB*, *B2M*, and *GAPDH*) as expected (**Supplementary Figure 1B**), and immune cells were specifically marked by *PTPRC*, confirming data quality (**Supplementary Figure 1C**). The integrated dataset showed homogeneous distribution across patients, suggesting successful batch effect correction (**Figure 1B**).

Based on canonical marker genes, we annotated these 18 clusters into six major cell lineages, including T/NK cells (6 clusters), myeloid cells (7 clusters), B cells (2 clusters), hepatocytes, endothelial cells, and hepatic stellate cells (HSCs) (**Figures 1C, D**). Immune cells, including T/NK, myeloid, and B cells, collectively accounted for over 70% of all profiled cells. Lineage-specific markers confirmed accurate annotation included T cells (*CD3E*, *CD3G*), NK cells (*KLRF1*, *KLRD1*), myeloid cells (*LYZ*, *C1QB*), B cells (*MS4A1*, *CD79A*), plasma cells (*IGLL1*, *MZB1*), hepatocytes (*ALB*, *APOA1*), endothelial cells (*PECAM1*, *CDH5*), and HSCs (*COL1A1*, *ACTA2*). All cell types were consistently detected across patients and tissue types, albeit with varying proportions (**Supplementary Figure 1D**).

We next compared the cell composition between A-HCC and NA-HCC, and between tumors and adjacent normal tissues (**Figures 1E, F**; **Supplementary Figure 1E**). Notably, both HCC subtypes exhibited hallmarks of an immunosuppressive tumor microenvironment. CD8^+^ T cells and NK/NKT cells were markedly depleted in tumors compared to adjacent normal tissues (82.43% vs. 43.93% and 69.08% vs. 29.40% of immune cells, respectively). Conversely, Tregs were substantially enriched in tumors, increasing from 0.81% in adjacent normal to 9.92% in A-HCC tumors, and from 1.48% to 6.39% in NA-HCC tumors. This is consistent with previous reports of Treg-mediated immunosuppression in HCC (18). Macrophages were also consistently increased in tumors relative to adjacent tissues in both HCC subtypes. Among non-immune populations, hepatocytes were the dominant cell type in tumor tissues. The proportions of major immune and non-immune cell populations in tumors and normal tissues across different patients were consistent with overall results (**Supplementary Figure 1E**). Taken together, these data suggest a shared immunosuppressive architecture in the tumor microenvironment of both A-HCC and NA-HCC, characterized by fewer cytotoxic lymphocytes and more immunosuppressive cells, such as Tregs and macrophages.

### A-HCC Tregs display a more potent immunosuppressive phenotype

**Figure 2:**
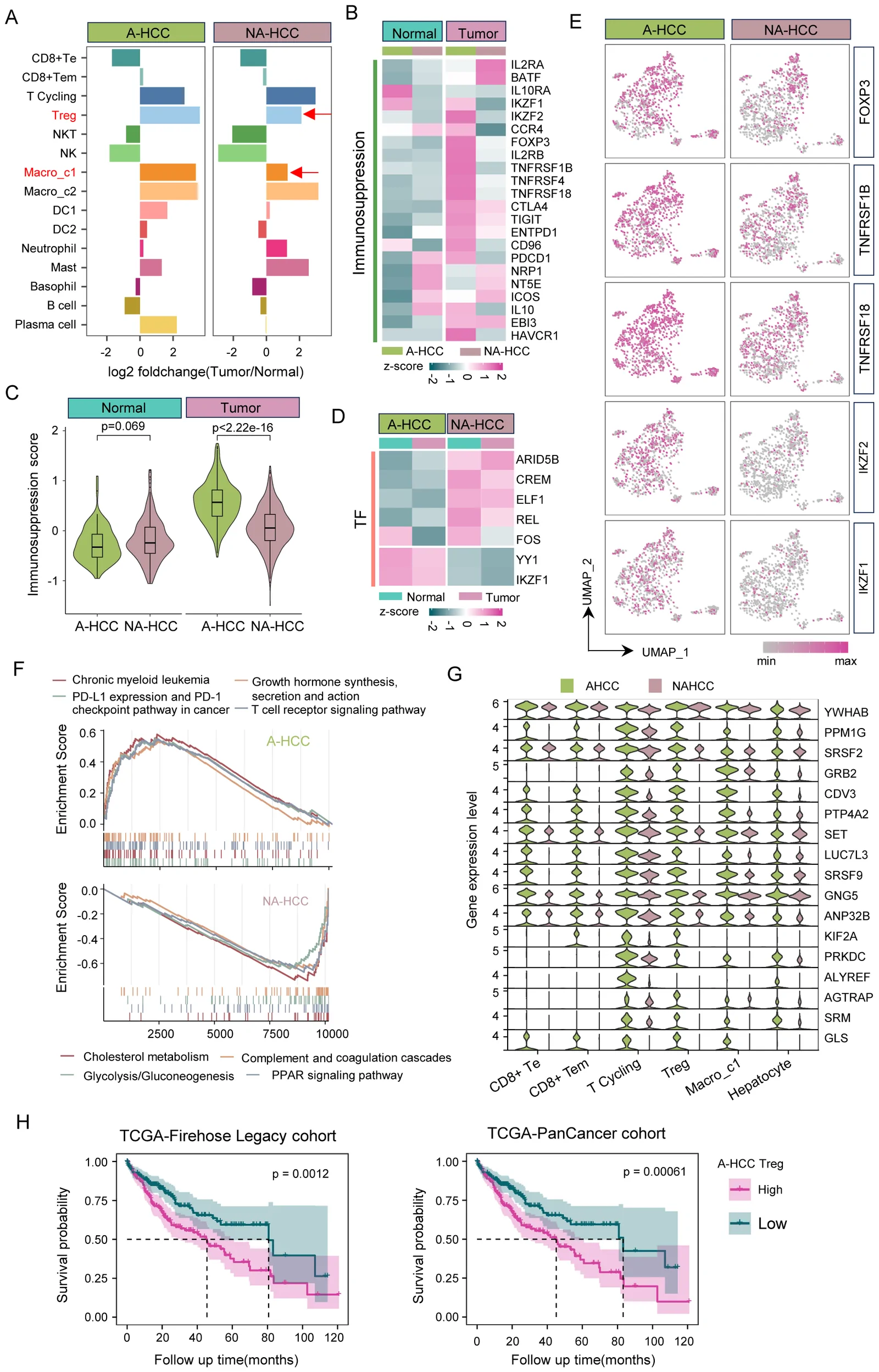
The Tregs exhibit a more immunosuppressive phenotype in A-HCC. **(A)** Fold changes in immune cell proportion (Tumor vs. Normal) in A-HCC and NA-HCC. **(B)** Heatmap of immunosuppressive signature gene expression in Tregs. Top bar: tissue type (Normal/Tumor); bottom bar: HCC subtype (A-HCC/NA-HCC). **(C)** Violin and box plots of immunosuppressive signature scores in Tregs of A-HCC and NA-HCC. Left: Normal; right: Tumor. p-values were calculated by two-tailed Wilcoxon rank-sum tests. **(D)** Heatmap of TF expression patterns in Tregs across HCC subtypes and tissue types. **(E)** UMAP plots showing expression of selected immunosuppressive genes in Tregs across two HCC subtypes. **(F)** GSEA analysis comparing tumor-derived Tregs between A-HCC and NA-HCC. Positive scores (top) indicate pathway enriched in A-HCC; negative scores (bottom) indicate enrichment in NA-HCC. **(G)** Violin plots of prognostic signature gene expression in tumor Tregs from A-HCC vs NA-HCC. **(H)** Kaplan-Meier curves for overall survival (OS) grouped by A-HCC Treg-derived signature score in two independent TCGA HCC cohorts (left: Firehose Legacy; right: PanCancer Atlas).

Both HCC subtypes exhibited an immunosuppressive tumor microenvironment, but we observed that Treg enrichment was more pronounced in A-HCC. Specifically, Tregs increased 12.3-fold in A-HCC tumor versus adjacent normal tissue, compared with 4.32-fold in NA-HCC (**Figure 2A**). This suggests a more immunosuppressive environment in alcohol-associated tumors. To assess the immunosuppressive function of Tregs, we examined the expression of immunosuppressive signature genes. Tregs from A-HCC tumors displayed significantly higher expression of genes encoding immunosuppressive mediators and regulators, including *IL10RA*, *IL2RB*, *IKZF1*, *IKZF2*, and *CCR4 etc.*, than those from NA-HCC tumors (**Figure 2B**). Consistent with that, an immunosuppression signature score was markedly increased in A-HCC tumor Tregs relative to that in NA-HCC tumor Tregs, while there was no significant difference in normal tissue Tregs (**Figure 2C**). Analysis of transcription factor (TF) expression revealed that A-HCC Tregs upregulated *YY1* and *IKZF1* (**Figure 2D**), the latter being a known regulator of Treg differentiation and suppressor function that promotes an immunosuppressive environment and impedes anti-tumor immunity (39). Together, these findings suggest that Tregs within A-HCC tumors may possess greater immunosuppressive potential, characterized by an expanded population and a heightened functional potency.

To determine whether this functional enhancement reflected shifts in Treg subpopulations, we performed sub-clustering analysis and identified four Treg clusters. However, the distribution of these subsets was similar between A-HCC and NA-HCC (**Supplementary Figures 2A, B**). Therefore, the observed functional differences are more likely attributable to transcriptional reprogramming rather than compositional shifts. Indeed, projection onto UMAP space revealed that key immunosuppressive genes were consistently upregulated across all Treg clusters in A-HCC compared with NA-HCC. (**Figure 2E**).

To functionally characterize Tregs, we performed differential expression and KEGG pathway enrichment analyses on Tregs from A-HCC versus NA-HCC tumors (**Figure 2F**; **Supplementary Figures 2C, D**). Notably, genes upregulated in A-HCC Tregs were significantly enriched in pathways related to immune regulation, such as the PD-1 pathway, T cell receptor signaling pathway, Rap1 signaling pathway, and long-term depression. In contrast, NA-HCC Tregs preferentially upregulated genes involved in metabolism, encompassing cholesterol metabolism, glycine/serine/threonine metabolism, glutathione metabolism, fructose/mannose metabolism, PPAR signaling, and glycolysis/gluconeogenesis. These findings indicated divergent regulatory programs. A-HCC Treg tended toward immunosuppressive pathways, whereas NA-HCC Tregs exhibit a predominantly metabolic signature.

As central mediators of immunosuppression, Tregs prompted us to hypothesize that genes upregulated in A-HCC tumor Tregs hold clinical prognostic significance. Univariate Cox regression analysis in two independent HCC cohorts (TCGA Firehose Legacy and TCGA PanCancer Atlas) identified a set of A-HCC Treg-upregulated genes significantly associated with overall survival (p < 0.01) (**Supplementary Figures 3A, B**). Notably, more than 66% of these prognosis-associated genes predicted worse prognosis in both cohorts. Furthermore, these poor-prognostic genes were not only upregulated in Tregs but also showed higher expression across other immune cell populations in A-HCC relative to NA-HCC (**Figure 2G**), suggesting a widespread immunosuppressive program. We then calculated a GSVA signature score based on these genes. Elevated signature scores were consistently associated with worse overall survival in both TCGA cohorts (**Figure 2H**).

To assess whether the prognostic value of the Treg signature is independent of major clinical covariates, we performed multivariate Cox regression analyses including age and sex as covariates in both TCGA cohorts. The Treg signature remained significantly associated with overall survival after adjusting for age and sex (p < 0.01 for both cohorts), whereas age and sex showed no significant association with prognosis (**Supplementary Table 3**). These results indicate that the prognostic value of the Treg signature is independent of these basic demographic factors. This coherent pattern is consistent with the conclusion that enhanced Treg-mediated immunosuppression in A-HCC promotes immune evasion and contributes to poorer patient prognosis. While these findings support the prognostic relevance of the A-HCC Treg signature, external validation in additional independent cohorts would be necessary to confirm its generalizability. Validation in future large-scale, well-annotated cohorts represents an important direction for follow-up studies.

### Anti-inflammatory macrophages dominate in A-HCC

In the HCC microenvironment, macrophages are known to acquire distinct polarization states. Inflammatory-associated polarization is linked to antitumor responses (40), whereas tumor-associated macrophages (TAMs) typically exhibit an immunosuppressive, pro-tumorigenic phenotype that promotes angiogenesis, tissue remodeling, invasion, and metastasis (41–43). To assess their main role in shaping the tumor immune landscape, we characterized the macrophage subsets in A-HCC and NA-HCC. Macrophage enrichment was strikingly higher in A-HCC tumors. Specifically, of the two major macrophage clusters identified, Macro_c1 was dramatically increased in tumors versus adjacent normal tissues by 10.9-fold in A-HCC compared to only 2.43-fold in NA-HCC. Similarly, Macro_c2 enrichment in tumor tissue was more pronounced in A-HCC (11.24-fold) than in NA-HCC (8.79-fold) (**Figure 2A**). These quantitative differences prompted us to further investigate their functional polarization states.

**Figure 3:**
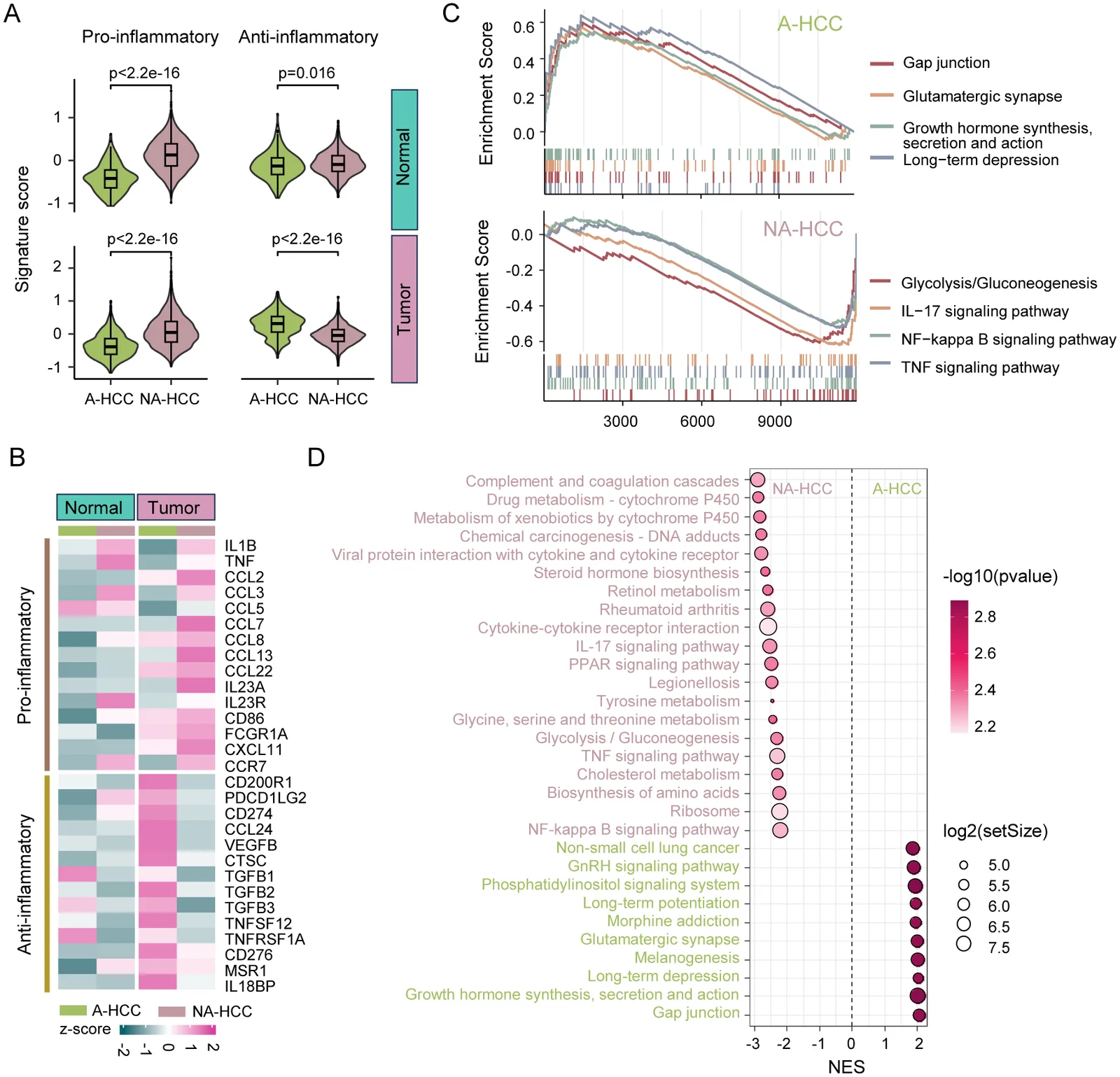
Functional features of Macrophage across two HCC types. **(A)** Violin and box plots of pro-inflammatory (left) and anti-inflammatory (right) signature scores in macrophages across A-HCC and NA-HCC. Top: Normal; bottom: Tumor. p-values were calculated by two-tailed Wilcoxon rank-sum tests. **(B)** Heatmap of pro-inflammatory and anti-inflammatory signature gene expression in macrophages. Top bar: tissue type (Normal vs. Tumor); bottom bar: HCC subtypes (A-HCC vs. NA-HCC). **(C)** GSEA comparing tumor-derived macrophages between A-HCC and NA-HCC. Positive enrichment scores (top) indicate pathways enriched in A-HCC; negative scores (bottom) indicate enrichment in NA-HCC. **(D)** Dot plots of significantly enriched KEGG pathways from GSEA comparing tumor-derived macrophages between A-HCC and NA-HCC. Positive NES (right dots) represents enrichment in A-HCC; negative NES (left dots) represents enrichment in NA-HCC. NES, normalized enrichment score.

We systematically evaluated macrophage functional polarization in both HCC subtypes by calculating the average expression of pro- and anti-inflammatory genes signature scores at single-cell resolution (**Figure 3A**; **Supplementary Figure 4A**). The analysis revealed that macrophages in A-HCC tumors were predominantly polarized toward an anti-inflammatory state, whereas those in NA-HCC tumors exhibited a pro-inflammatory profile. The difference was also observed, though less pronounced, in adjacent normal tissues. At the gene level, pro-inflammatory markers (eg., *IL1B*, *TNF*, *IL23*, and *CCR7*) were increased in NA-HCC tumor macrophages, while anti-inflammatory genes (eg., *CD274*/*PD-L1*, *CCL24*, *CTSC*, *TGFB2*, and *TNFSF12*) were more highly expressed in A-HCC tumor macrophages (**Figure 3B**). Together, these findings suggest a fundamental difference in macrophage polarization between the two HCC subtypes. NA-HCC appears to be characterized by a pro-inflammatory tumor microenvironment that may amplify immune responses, while A-HCC may be dominated by an anti-inflammatory microenvironment that likely facilitates immune evasion and supports tumor progression.

We next extended our functional analysis to macrophages via differential expression and KEGG pathway enrichment analyses comparing A-HCC and NA-HCC tumors (**Figures 3C, D**; **Supplementary Figure 4B**). Intriguingly, the pathways enriched in A-HCC macrophages showed patterns remarkably similar to those observed in Tregs, including gap junction, cholinergic synapse, glutamatergic synapse, GnRH signaling pathway, and long-term depression. Conversely, NA-HCC macrophages shared a propensity for metabolic pathways (cholesterol metabolism, glutathione metabolism, glycolysis/gluconeogenesis, and PPAR signaling pathway) with NA-HCC Tregs, while also uniquely enriching the pro-inflammatory IL-17 signaling pathway. These findings suggest that A-HCC and NA-HCC harbor fundamentally different tumor microenvironments. The A-HCC microenvironment appeared to be characterized by anti-inflammatory reprogramming of both lymphoid (Treg) and myeloid (macrophage) lineages, whereas the NA-HCC microenvironment was predominantly pro-inflammatory and metabolically active.

### Distinct CD8^+^ T/NK cell dysfunction and metabolism in A-HCC versus NA-HCC

As cytotoxic T lymphocytes (CTLs), CD8^+^ T cells are central mediators of adaptive anti-tumor immunity. They recognize tumor-associated antigens presented by major histocompatibility complex class I (MHC I) and eliminate target cells by releasing perforin and granzymes. This response is complemented by innate cytotoxic lymphocytes NK and NKT cells, which provide critical immunosurveillance through direct tumor cell recognition and killing. To assess their combined role in tumor control across distinct tumor microenvironments, we next characterized the functional state of these cytotoxic lymphocytes in A-HCC versus NA-HCC.

Consistent with our earlier observations of immune cell distribution (**Figures 1E, F**), tumor tissues in both A-HCC and NA-HCC were dominated by immunosuppressive populations, such as Tregs, macrophages, and cycling T cells. Conversely, adjacent normal tissues showed a relative enrichment of CD8^+^ T cells and NK/NKT cells. Further quantitative analysis revealed a consistent and significant depletion of effector CD8^+^ T cells (CD8+ Te) and NK/NKT cells in tumors compared to peritumoral regions across both subtypes (**Figure 2A**). Together with the concurrent enrichment of Tregs, this depletion contributes to a profoundly immunosuppressive microenvironment that favors tumor progression.

**Figure 4:**
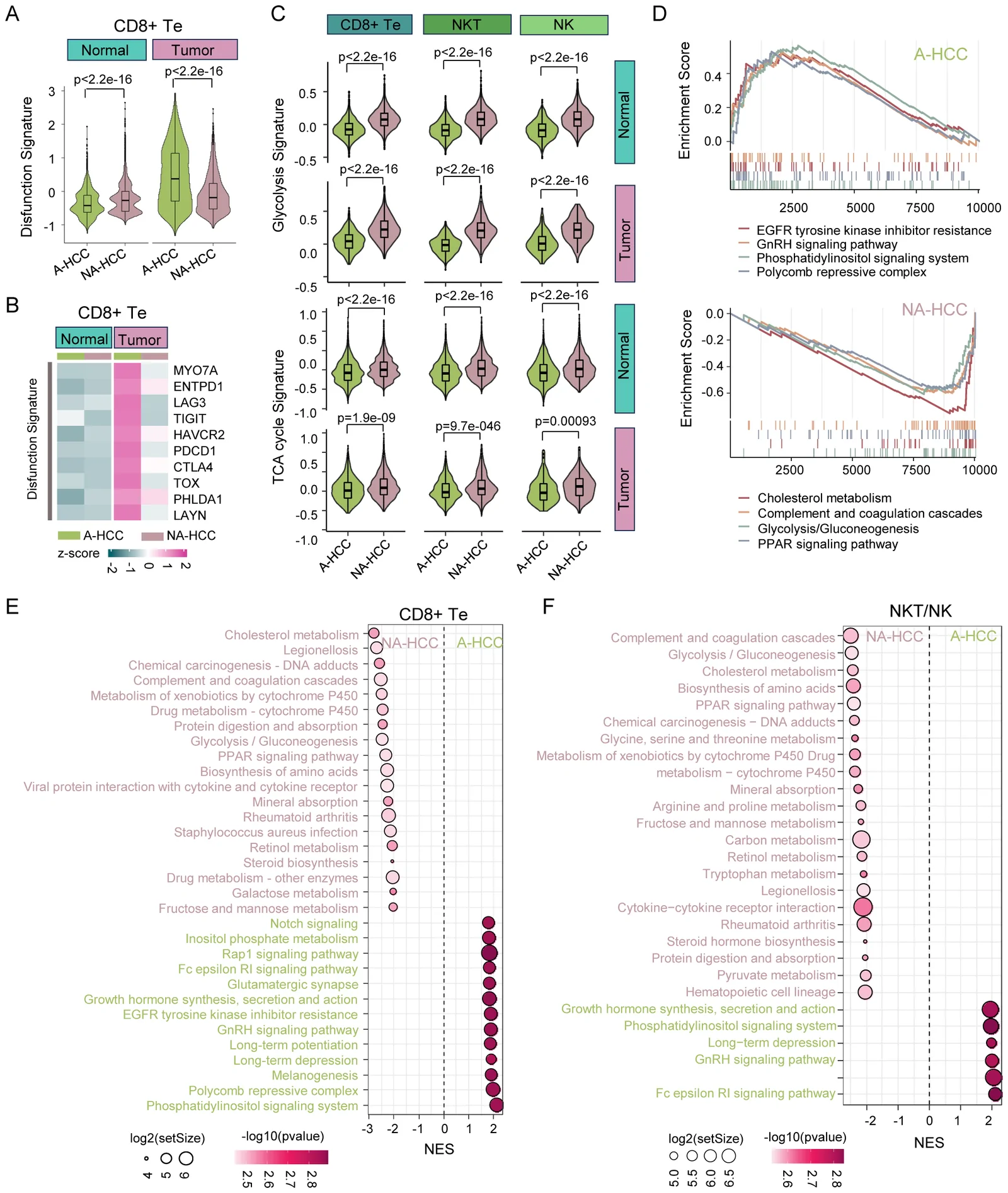
Characteristics of CD8^+^ effector T and NK/NKT cells across two HCC subtypes. **(A)** Violin and box plots of dysfunction signature scores in CD8^+^ effector T (CD8^+^ Te) cells across A-HCC and NA-HCC. Left: Normal; right: Tumor. p-values were calculated by two-tailed Wilcoxon rank-sum tests. **(B)** Heatmap of dysfunction signature gene expression in CD8^+^ Te cells. Top bar: tissue type (Normal vs. Tumor); bottom bar: HCC subtype (A-HCC vs. NA-HCC). **(C)** Violin and box plots of glycolysis and TCA cycle signature scores in CD8^+^ Te, NK and NKT cells across two tissues and two HCC subtypes. p-values were calculated by two-tailed Wilcoxon rank-sum tests. **(D)** GSEA analysis comparing tumor-derived CD8^+^ Te cells between A-HCC and NA-HCC. Positive enrichment scores (top) indicate pathways in A-HCC; negative scores (bottom) indicate enrichment in NA-HCC. **(E)** Dot plots of significantly enriched KEGG pathways from GSEA of tumor-derived CD8^+^ Te cells. Positive NES (right dots) represents enrichment in A-HCC; negative NES (left dots) represents enrichment in NA-HCC. NES, normalized enrichment score. **(F)** Dot plots of significantly enriched KEGG pathways from GSEA of tumor-derived NK/NKT cells (layout and NES as in E).

To assess the functional state of CD8+ Te cells, we calculated a dysfunction signature score at single-cell resolution (**Figure 4A**). In both A-HCC and NA-HCC, tumor-infiltrating CD8+ Te cells showed higher dysfunction scores than those in peritumoral tissues. Notably, dysfunction was significantly more pronounced in A-HCC than in NA-HCC tumors, indicating a more severe impairment of cytotoxic function in alcohol-associated disease. Consistent with this heightened dysfunction, canonical inhibitory receptors (eg., *PDCD1*, *LAG3*, *CTLA4*, *TIGIT*, *LAYN*, and *HAVCR2*) were more highly expressed in A-HCC CD8+ Te cells (**Figure 4B**). This apparent discrepancy suggested that while NA-HCC CD8^+^ T cells exhibit a “classical exhausted” phenotype (high inhibitory receptor expression), A-HCC CD8^+^ T cells may undergo alternative or more profound dysfunction that extends beyond these canonical inhibitory markers.

Analysis of energy metabolism pathways revealed further divergence. Notably, NA-HCC showed significantly higher enrichment scores for glycolysis and the tricarboxylic acid (TCA) cycle in both tumor and peritumoral CD8+ Te cells and NK/NKT cells (**Figure 4C**), suggesting that CD8^+^ T and NK/NKT cell function in NA-HCC is more dependent on aerobic glycolysis which is a metabolic state associated with effector function and proliferation. In contrast, CD8+ Te and NK/NKT cells in A-HCC exhibited lower metabolic activity, consistent with their more severely dysfunctional phenotype.

Subsequent differential expression and KEGG pathway enrichment analyses were performed to compare CD8+ Te cells isolated from A-HCC and NA-HCC tumors (**Figures 4D, E**). Genes upregulated in A-HCC CD8+ Te cells showed significant enrichment in pathways related to glutamatergic synapse, GnRH signaling pathway, long-term potentiation, and sphingolipid signaling pathway. The profile is highly consistent with those previously identified in A-HCC Tregs and macrophages. In contrast, CD8+ Te cells from NA-HCC displayed upregulation of genes involved in major metabolic processes, including cholesterol metabolism, glutathione metabolism, glycolysis/gluconeogenesis, and PPAR signaling, again mirroring metabolic signatures observed in other NA-HCC immune subsets. Importantly, KEGG analysis of NK/NKT cells revealed an almost identical pattern of pathway enrichment (**Figure 4F**), suggesting that these transcriptional signatures may be not cell-type restricted but rather reflect broader, etiology-driven regulatory programs.

In summary, these data point to a fundamental distinction in the cytotoxic lymphocyte compartment between A-HCC and NA-HCC. A-HCC appears to be characterized by severe dysfunction of CD8+ Te and NK/NKT cells, higher expression of classical exhaustion markers, and enrichment of synapse-related and signaling pathways. By contrast, NA-HCC harbors CD8^+^ T and NK/NKT cells with lower inhibitory receptor expression and enhanced metabolic activity, notably aerobic glycolysis, suggesting a metabolically active effector state with potentially preserved functionality.

### Malignant hepatocytes in A-HCC exhibit enhanced stemness, EMT, and drug resistance score

Our analyses suggested that A-HCC exhibits a more profoundly immunosuppressive microenvironment compared with NA-HCC, characterized by stronger Treg-mediated suppression, anti-inflammatory macrophage polarization, and more severe dysfunction of CD8+ Te cells. Based on these observations, we hypothesized that the malignant hepatocytes in A-HCC might likewise acquire more aggressive biological features, potentially explaining its association with poorer clinical outcomes.

**Figure 5:**
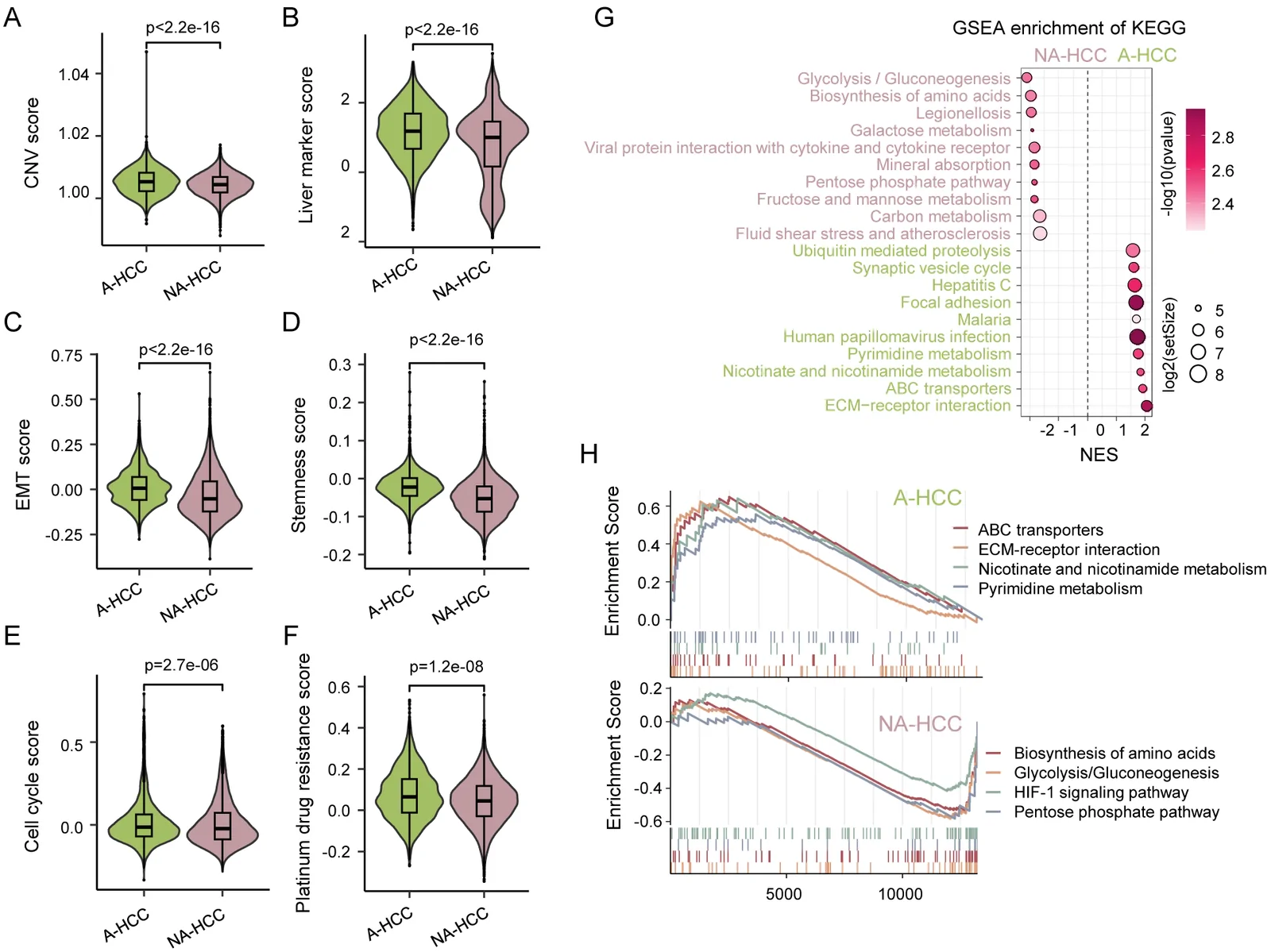
Characteristics of hepatocyte cells across two HCC subtypes. **(A)** Violin and box plots of CNV scores in tumor hepatocytes from A-HCC and NA-HCC. **(B)** Violin and box plots of liver marker scores (malignancy signature). **(C)** Violin and box plots of EMT scores. **(D)** Violin and box plots of stemness scores. **(E)** Violin and box plots of cell cycle scores. **(F)** Violin and box plots of platinum drug resistance scores. All p-values were calculated by two-tailed Wilcoxon rank-sum tests. **(G)** Dot plots of significantly enriched KEGG pathways from GSEA comparing tumor hepatocytes between A-HCC and NA-HCC. Positive NES (right dots) indicates enrichment in the A-HCC; negative NES (left dots) indicates enrichment in NA-HCC. NES, normalized enrichment score. **(H)** GSEA enrichment analysis comparing tumor hepatocytes cells between A-HCC and NA-HCC. Positive enrichment scores (top) indicate pathways in A-HCC; negative scores (bottom) indicate enrichment in NA-HCC.

To evaluate the malignant potential of hepatocytes, we first applied inferCNV analysis to infer large-scale chromosomal copy-number variations (CNV) from transcriptomic profiles. Compared with reference immune cells (T cells and myeloid cells), hepatocytes exhibited substantial CNVs confirming their malignant transformation (**Supplementary Figure 5**). Notably, the average CNV score per cell was significantly higher in A-HCC compared with NA-HCC hepatocytes (**Figure 5A**), indicative of heightened genomic instability and a more advanced malignant state in the alcohol-associated tumors. Consistent with this, an independent liver malignant signature also showed significantly higher scores in A-HCC hepatocytes (**Figure 5B**). Together, these integrated analyses suggest that hepatocytes within A-HCC may possess a more aggressive and genomically unstable malignant phenotype than those in NA-HCC.

We next investigated whether the observed increase in malignant potential correlates with functional hallmarks of aggressive tumor biology. Analysis using a well-established epithelial-to-mesenchymal transition (EMT) gene signature suggested significantly upregulation of EMT program in A-HCC hepatocytes, a process conferring enhanced motility and invasiveness (**Figure 5C**). Correspondingly, both stemness signature scores and cell cycle-related gene signature scores were elevated in A-HCC relative to NA-HCC (**Figures 5D, E**). These findings suggest that A-HCC tumors may be enriched in malignant hepatocytes possessing progenitor-like properties and augmented proliferative capacity.

Since aggressive tumor features frequently coincide with increased therapeutic resistance, we next evaluated drug resistance potential using KEGG platinum drug resistance gene set. Hepatocytes in A-HCC showed significantly higher platinum drug resistance associated gene signature scores than those in NA-HCC (**Figure 5F**), suggesting that A-HCC tumors may have enhanced expression of drug resistance related programs, though functional validation is needed to confirm this inference.

Substantial inter-patient heterogeneity in transcriptional programs of cancer cells has been reported (44). To dissect the molecular pathways underlying the aggressive phenotype of A-HCC hepatocytes, we performed differential expression analysis followed by KEGG pathway enrichment analyses comparing A-HCC and NA-HCC tumor hepatocytes (**Figures 5G, H**). Genes significantly upregulated in A-HCC were enriched in processes promoting invasion and metastasis (ECM-receptor interaction), chemotherapy resistance (ABC transporters), and nucleotide metabolism (nicotinate/nicotinamide metabolism, pyrimidine metabolism). Conversely, genes upregulated in NA-HCC were predominantly enriched in metabolic pathways such as glycolysis/gluconeogenesis, pentose phosphate pathway, biosynthesis of amino acids, and the metabolic master regulator HIF-1 signaling pathway. These pathway-level differences provide mechanistic explanation for the aggressive traits observed in A-HCC. Enrichment of ECM-receptor interactions may enhance invasive and metastatic potential, while overexpression of ABC transporters could actively efflux chemotherapeutic agents, thereby contributing to intrinsic drug resistance, though this requires experimental confirmation. In contrast, the metabolic pathway enrichment in NA-HCC, including glycolysis/gluconeogenesis, pentose phosphate pathway, amino acid biosynthesis, and HIF-1 signaling, suggested a predominant metabolic dependency on glycolytic and anabolic processes.

Taken together, our multidimensional analysis suggests that A-HCC may be characterized not only by a more immunosuppressive microenvironment but also by hepatocytes that possess intrinsically malignant features, including enhanced EMT, stemness, proliferative activity, and drug resistance, along with coordinated activation of pathways linked to invasion and treatment resistance. This dual aggressiveness at both the immune and tumor cell levels likely contribute to the poorer prognosis in A-HCC patients.

### Distinct ligand-receptor interactions are inferred to reinforce immunosuppression A-HCC

Integrative multi-lineage profile sugeeested that A-HCC may be characterized by coordinated reprogramming across immune and malignant cell population, including intensified Treg-mediated suppression, a shift toward anti-inflammatory macrophage phenotypes, severe CD8^+^ T cell dysfunction, and more aggressive hepatocytes. To map the intercellular signaling landscape underlying this reconfigured tumor microenvironment, we performed a systematically comparative analysis of cell-cell communication networks in A-HCC versus NA-HCC using ligand-receptor interaction analysis.

Global comparison of inferred interaction strengths showed that both the number and intensity of intercellular communications were reduced in A-HCC versus NA-HCC (**Supplementary Figure 6A**). The most frequent interactions occurred between hepatocytes and macrophages. Their prominent involvement in both outgoing and incoming interaction appeared to establish this cellular axis as a central communication hub in the A-HCC (**Supplementary Figure 6B**).

**Figure 6:**
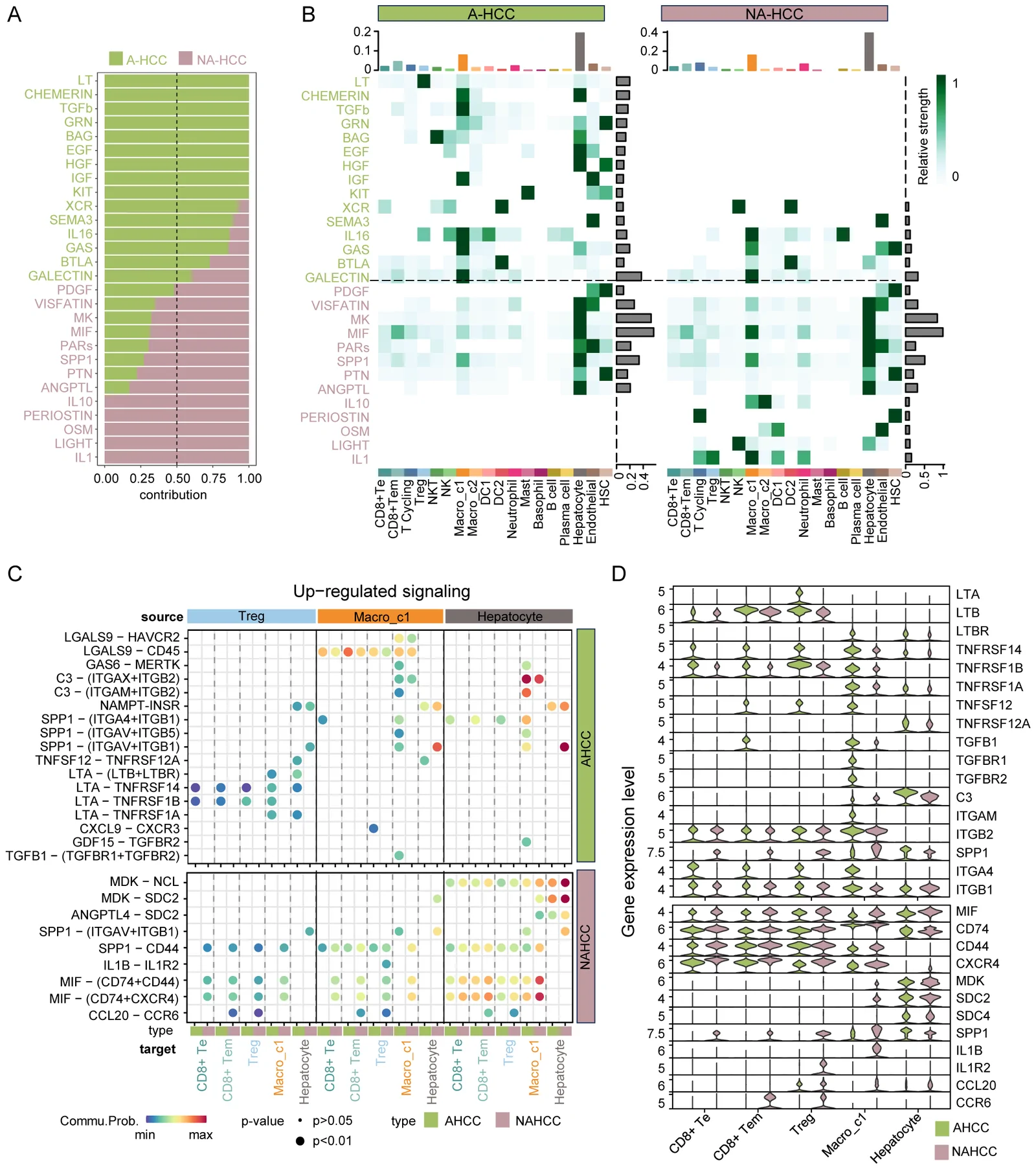
Comparison of Cell-Cell communication between A-HCC and NA-HCC. **(A)** Bar plot of context-specific signaling pathways ranked by differences in overall information flow between A-HCC and NA-HCC. **(B)** Heatmap of aggregated outgoing and incoming signaling for significantly different pathways between A-HCC and NA-HCC. **(C)** Dot plots showing communication probabilities of upregulated ligand-receptor pairs among Treg, Macro_c1, hepatocytes, CD8+ Te and CD8+ Tem. Top: upregulated ligand-receptor pairs in A-HCC; bottom: upregulated ligand-receptor pairs in NA-HCC. **(D)** Violin plots of upregulated signaling-related gene expression in CD8+ Te, CD8+ Tem, Treg, Macro_c1 and hepatocyte subsets across A-HCC and NA-HCC.

We next quantified the information flow of each signaling pathway to identify etiology-specific communication patterns (**Figures 6A, B**). The A-HCC interactome appeared to be characterized by pathways conducive to immunosuppression and tumor promotion, notably including lymphotoxin (LT), CHEMERIN, transforming growth factor beta (TGF-β), and granulin (GRN) signaling. In contrast, the NA-HCC network exhibited significant enrichment of pathways associated with pro-inflammatory signaling and immune activation, such as IL-1, LIGHT, oncostatin M (OSM), PERIOSTIN, and IL-10 signaling.

To elucidate the molecular basis of the identified pathway alterations, we performed a comparative analysis of ligand-receptor interaction probabilities between the two HCC types (**Figure 6C**). In A-HCC, the upregulation of specific interactions provided potential molecular correlates for the observed cellular phenotypes. Enhanced LTA-mediated signaling through receptors on Tregs, CD8^+^ T cells, macrophages, and hepatocytes may foster the immunosuppressive microenvironment. The upregulated LGALS9-HAVCR2 interaction is inferred to connect anti-inflammatory macrophages to impaired CD8^+^ T cell function. Elevated GAS6-MERTK and TGF-β1 signaling may contribute to the observed M2-like macrophage polarization and heightened Treg suppression, respectively. By contrast in NA-HCC, the upregulation of pro-inflammatory interactions, such as MIF and IL1B, is consistent with its inflammatory microenvironment. Subsequent gene expression analysis validated these interaction patterns, showing significant upregulation of key immunosuppressive mediators (e.g., *LTA*, *TGFB1*) in A-HCC, whereas pro-inflammatory and metabolic regulators (e.g., *MIF*, *IL1B*) were elevated in NA-HCC (**Figure 6D**).

Together, our cell-cell communication analysis suggests that the immunosuppressive microenvironment in A-HCC is not simply an aggregate of individual cellular changes, but may be a coordinated network actively reinforced by specific ligand-receptor interactions. The enhanced activity of the LTA, LGALS9-HAVCR2, GAS6-MERTK, and TGF-β signaling pathways provides a molecular framework that unifies our observations across Tregs, macrophages, CD8^+^ T cells, and hepatocytes into a coherent model of A-HCC microenvironment. This interconnected immunosuppressive network is inferred to contribute to the aggressive clinical behavior of A-HCC and may inform the development of etiology-specific therapies, though these ligand-receptor interactions are inferred from gene expression and require experimental validation.

### Sensitivity analyses validate the robustness of key findings

So far, our results suggested that A-HCC has a distinct immunosuppressive network. But we wanted to check whether these conclusions might be influenced by integrated method, the imbalance in sample sizes, or the way we handled single cell statistical inference. So, we carried out three sensitivity analyses.

To assess whether our conclusions are dependent on the specific integration method, we repeated the main analyses using Harmony-integrated data (**Supplementary Figures 7A–C**). Although CD8^+^ Te and NK cells did not separate cleanly during clustering in the Harmony-integrated dataset, the key findings that were robustly reproducible. Treg and macrophage enrichment was more pronounced in A-HCC tumors (**Supplementary Figure 7D**). Treg immunosuppression scores, and macrophage pro-inflammatory and anti-inflammatory scores showed consistent with those obtained from CCA-integrated data (**Supplementary Figures 7E, F**). These results confirm that our conclusions regarding Treg and macrophage biology are robust to integration method choice.

To exclude the possibility that the imbalanced sample size (2 A-HCC vs. 7 NA-HCC) drives the observed differences, we performed a random subsampling analysis. From the seven NA-HCC samples, we randomly selected two samples (matching the number of A-HCC samples) and recalculated the tumor-to-normal fold change of Treg proportions, and repeated this 20 times. As shown in **Supplementary Figure 8A**, the Treg fold change for NA-HCC across all iterations ranged from 4.2 to 5.1, consistently lower than the 12.3-fold change observed in A-HCC. So, the enhanced Treg enrichment in A-HCC dose not appear to be an artifact of unbalanced group size.

Finally, to avoid overinterpreting single cell level statistical significance as independent biological replication, we performed pseudobulk analyses by aggregating gene expression counts per patient and cell type. Key findings were re-evaluated at the patient level, including cell type proportion fold changes, Treg immunosuppression scores, macrophage polarization scores, and CD8^+^ T/NK dysfunction scores. The trends matched what we saw at the single-cell level (**Supplementary Figures 8B–D**), though some comparisons lost statistical significance. Pseudobulk analyses effectively reduce the sample size to the number of patients, so the statistical power drops considerable. Both Zimmerman et al. and Squair et al. have discussed this trade-off that pseudobulk approaches are more conservative but less powered when the number of biological replicates is small (45, 46).

We extended the pseudobulk approach to the ligand-receptor genes that came out of the cell-cell communication analysis. We checked the expression of the key genes involved in the inferred signaling pathways across the main cell types (Tregs, CD8^+^ T cells, macrophages, and hepatocytes). The pseudobulk expression trends were consistent with what we had seen at the single-cell level. Genes related with LTA, LGALS9-HAVCR2, GAS6-MERTK and TGF-β1 mediated signaling were higher in A-HCC (**Supplementary Figure 9A**), while genes related with MIF and IL1B mediated signaling were elevated in NA-HCC (**Supplementary Figure 9B**). As with the other pseudobulk analyses, some of these differences did not reach statistical significance, again due to the limited number of biological replicates. But the directional consistency across all the genes tested gave us more confidence that the inferred communication differences between A-HCC and NA-HCC are not simply a byproduct of the single-cell analytical framework.

Taken together, these sensitivity analyses show that our main findings, including Treg enrichment, macrophage polarization, CD8^+^ T and NK/NKT cell dysfunction, and the inferred ligand-receptor communication patterns, are robust to integration method, cohort composition, and analytical strategies. They are not artifacts of any single methodological choice.

## Discussion

Although alcohol consumption is a well-established global risk factor for HCC, A-HCC remains poorly characterized compared with NA-HCC (24). Our study provides the first single-cell transcriptomic profile of human A-HCC and a direct comparison with NA-HCC. As an exploratory resource, the profile establishes a foundational framework for understanding the cellular and molecular basis of A-HCC and serves as a critical reference for future large-scale validation and mechanistic studies. The main findings include that (i) A-HCC may have a more immunosuppressive microenvironment than NA-HCC, driven by higher Treg enrichment and function, anti-inflammatory macrophages, and more severe CD8^+^ T cell dysfunction; (ii) A-HCC hepatocytes are more malignant, showing higher CNV, EMT, stemness, proliferation, and drug resistance score; (iii) specific ligand-receptor interactions (LT, LGALS9-HAVCR2, GAS6-MERTK, TGF-β) may reinforce a coordinated immunosuppressive network.

Both HCC subtypes share common immunosuppressive features, such as Treg and macrophage accumulation and CD8^+^ T cell depletion. However, the magnitude is clearly greater in A-HCC. Tregs in A-HCC not only increase in number but also upregulate immunosuppressive genes (*IKZF1*, *YY1*, *IL10RA*, *CCR4*) and show higher pathway activity (PD-1, TCR signaling). Moreover, a Treg-derived gene signature predicted worse survival in two independent cohorts, supporting the clinical relevance of this enhanced suppression. Macrophages in A-HCC shift strongly toward an anti-inflammatory phenotype, with high expression of *CD274* (*PD-L1*), *TGFB2* and other M2-like genes, whereas NA-HCC macrophages are more pro-inflammatory. This is consistent with the known ability of chronic alcohol exposure to polarize macrophages toward a pro-tumorigenic state (47, 48). CD8^+^ T cells in A-HCC show not only higher expression of classic exhaustion markers but also a more severe dysfunction score and lower metabolic activity (glycolysis, TCA cycle). The higher inhibitory receptors yet lower metabolic activity suggests A-HCC CD8^+^ T cells may enter a deeper or alternative exhaustion state that is less responsive to checkpoint blockade. It may have therapeutic implications that NA-HCC might respond better to PD-1/PD-L1 inhibitors because its T cells are “metabolically active” but exhausted, while A-HCC may need combination strategies that also modulate Tregs or metabolism.

Beyond the immune cells, malignant hepatocytes themselves are more aggressive in A-HCC. They have higher CNV scores, increased EMT, stemness, and proliferative capacity, and they showed enhanced platinum drug resistant score. Pathway enrichment analysis revealed that A-HCC hepatocytes preferentially upregulate pathways involved in ECM-receptor interaction and ABC transporters. ABC transporter enrichment is a known resistance mechanism in HCC (49, 50). The increased related-genes signature scores and enriched pathways suggest a potential mechanism, but direct functional validation is needed to confirm. In contrast, NA-HCC hepatocytes rely more on glycolysis, pentose phosphate, and HIF-1 signaling. Glycolysis and lipid Metabolic reprogramming is a hallmark of HCC enabling tumor cells adaptation and proliferation (51, 52). These findings suggest that the two etiological subtypes drive distinct metabolic and invasive programs. Of particular clinical relevance may be the enhanced drug resistance signature score in A-HCC, which could underlie the frequently poor response of these patients to conventional chemotherapy regimens. Mechanistically, persistent alcohol-induced oxidative stress and cumulative DNA damage are likely contributors to increased genomic instability (reflected in higher CNV) and may favor the selection of more aggressive cellular clones.

The functional alterations observed across distinct cell types do not occur in isolation. Instead, they appear to be integrated through specific ligand-receptor interactions that shape the tumor ecosystem. Cell-cell communication analysis suggested that A-HCC is enriched with immunosuppressive signaling pathways (LT, CHEMERIN, TGF-β, GRN) while pro-inflammatory pathways (IL-1, LIGHT, OSM, PERIOSTIN) are downregulated compared with NA-HCC. LGALS9-HAVCR2 has been reported to promote CD8^+^ T cell exhaustion in HCC and other solid tumors (53, 54). GAS6-MERTK signaling has been implicated in HCC progression and immune evasion (55, 56). TGF-β signaling is a well-established master regulator of immunosuppression (57, 58). LGALS9-HAVCR2 between macrophages and T cells is reported to inhibit T cell function, linking anti-inflammatory macrophages to CD8^+^ T cell dysfunction. GAS6-MERTK and TGF-β1 may further polarize macrophages toward an anti-inflammatory state and promote Treg differentiation. LTA/LTB signaling, enriched in multiple cell types, may help organize tertiary lymphoid structures that contribute to local immunosuppression. In NA-HCC, by contrast, pro-inflammatory signals (MIF, IL1B) dominate, maintaining a more metabolically active and less suppressed environment. These differences suggest that targeting communication pathways (e.g., blocking LGALS9-HAVCR2 or TGF-β) could be more effective in A-HCC than in NA-HCC. Indeed, targeting these intercellular communication pathways, either through ligand/receptor blockade or modulation of downstream signaling, has emerged as a promising strategy in HCC (59, 60). These ligand-receptor interactions are inferred from gene expression and require orthogonal experimental validation.

We also performed sensitivity analyses, including Harmony integration, random subsampling, and pseudobulk aggregation extended to ligand-receptor genes, to assess the robustness of our findings. The main results remained consistent across all approaches, suggesting that our conclusions are not driven by integration method, cohort imbalance, or single-cell-level pseudoreplication.

Several limitations of this study should be noted. First, the A-HCC cohort is limited to only two patients, both male and form a single institution, which restricts the generalizability of our findings. This small sample size makes it difficult to exclude patient-level variation, sex differences or institutional biases. While obtaining fresh A-HCC surgical specimens is inherently challenging and no prior single-cell data exist for this etiology, this study should be interpreted as an exploratory resource and hypothesis generator rather than as definitive evidence. Future studies with larger, multi-center, sex-balanced cohorts are needed for validate. Second, although we performed batch correction using CCA validated by LISI evaluation, the use of a public NA-HCC dataset may still introduce batch effects and cohort-specific confounders, including differences in clinical stage, cirrhosis, viral status and sample processing. While our integration and sensitivity analyses support the robustness of our major findings, residual confounding cannot be fully excluded. Third, our conclusions are based on transcriptomic data and computational inference rather than direct functional measurements. Thus, experimental validation using multiplex immunofluorescence, co-culture systems, or spatial transcriptomics is needed to confirm these inferred interactions and establish causality, such as the role of LGALS9-HAVCR2 in T cell exhaustion. Finally, the Treg-derived prognostic signature requires further validated in larger, prospective cohorts with comprehensive clinical annotation.

Despite these limitations, this study represents the first comprehensive single-cell atlas of A-HCC and provides a systematic comparison with NA-HCC. The convergence of multiple lines of evidence, across cell type composition, functional signatures, and inferred communication networks, supports the view that A-HCC and NA-HCC have fundamentally different immune ecosystems. Our findings serve as a foundational resource and generate testable hypotheses for future mechanistic and translational studies.

Our findings highlight that A-HCC is not simply a subset of HCC with a similar immune landscape. It may have a distinct, more aggressive biology involving both immune and tumor cells. This may have at least three clinical implications. First, immunotherapy strategies that work well in NA-HCC (e.g., anti-PD-1) might be less effective in A-HCC due to the deeper CD8^+^ T cell dysfunction and stronger Treg suppression. Combining checkpoint inhibitors with Treg-depleting agents or macrophage-modulating drugs could be explored. Second, the higher drug resistance score in A-HCC hepatocytes may suggest that chemotherapy alone could be insufficient; targeting ABC transporters or using alternative drug combinations might improve outcomes. Third, the communication pathways we identified (LGALS9-HAVCR2, GAS6-MERTK, TGF-β) are druggable and could be tested in A-HCC-specific preclinical models.

**Figure 7:**
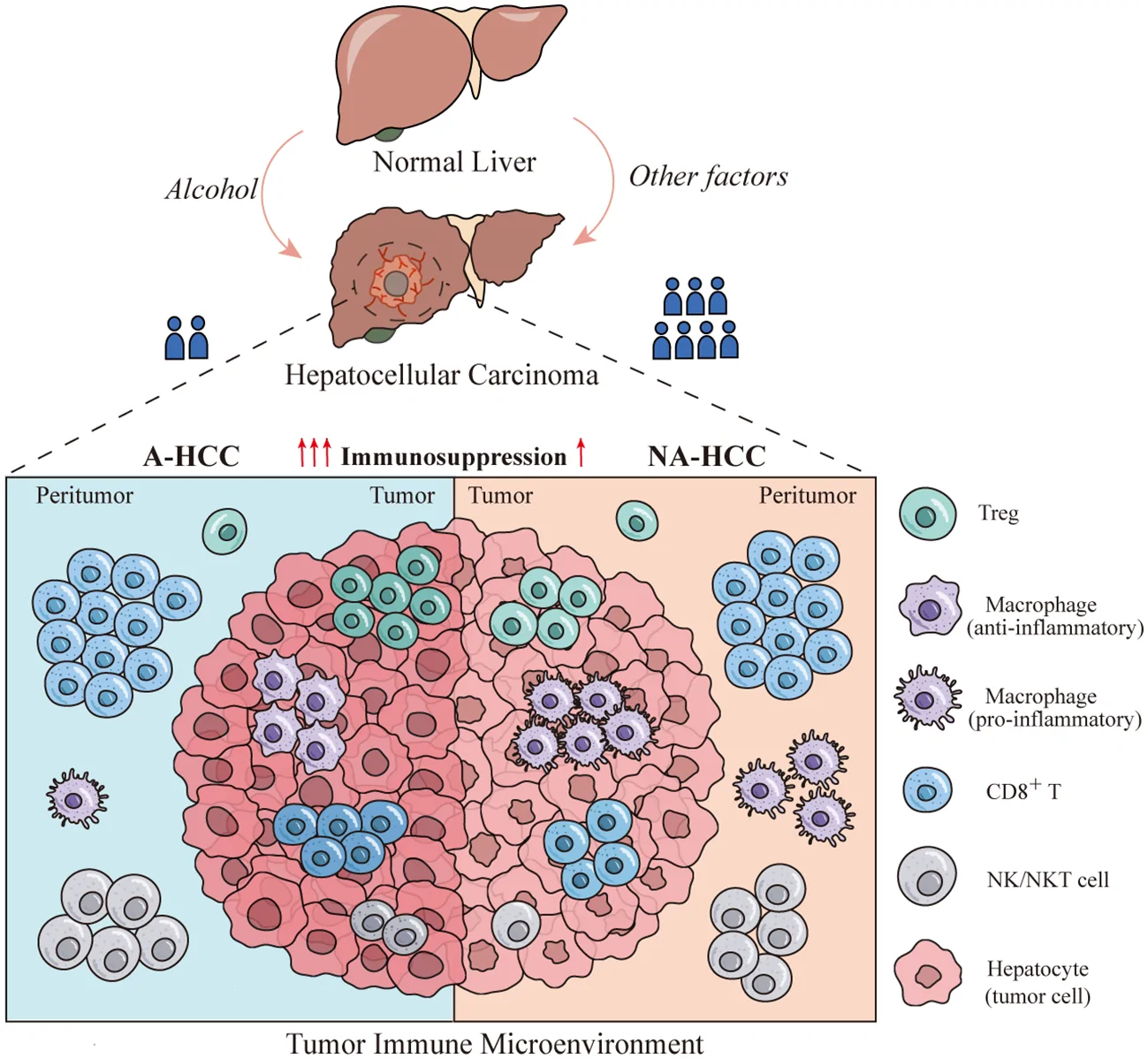
Schematic overview of TME heterogeneity between A-HCC and NA-HCC. A-HCC displays a coordinated immunosuppressive and malignant ecosystem that distinguished it form NA-HCC, driven by enhanced Treg suppression, anti-inflammatory macrophage, CD8^+^ T cell and NK/NKT dysfunction, and aggressive hepatocytes.

In summary, we present the first single-cell transcriptomic profile of A-HCC and a systematic comparison with NA-HCC. A-HCC appears to be characterized by a coordinated immunosuppressive ecosystem with enhanced Treg and anti-inflammatory macrophage activity coupled with severe CD8^+^ T cell dysfunction, plus intrinsically more malignant hepatocytes (**Figure 7**). These are linked by specific intercellular communication networks. Our results provide a molecular framework for understanding the poor prognosis of A-HCC and point to potential etiology-specific therapeutic targets. As the first comprehensive single-cell resource for A-HCC, this study not only advances our understanding of the aggressive biology of alcohol-associated liver cancer but also serves as a foundational platform for future large-scale validation, mechanistic dissection, and the development of etiology-tailored therapeutic strategies.

## Data Availability

The scRNA-seq raw data have been deposited in the Genome Sequence Archive (GSA) with accession number HRA017333, and are available from the corresponding author upon reasonable request.

## Supplementary material

Supplementary Figure 1 ScRNA-seq analysis of the TME in A-HCC and NA-HCC. **(A)** Bar plot showing Local Inverse Simpson’s Index (LISI) scores for five integration methods. **(B)** UMAP plots showing expression of housekeeping genes across major cell types. **(C)** UMAP plot showing expression of *PTPRC* across major cell types. **(D)** UMAP plots showing distribution of cell subtypes in normal and tumor samples from A-HCC and NA-HCC patients. **(E)** Bar plots showing proportion of immune and non-immune cell populations in tumor and normal tissues across individual patients.

Supplementary Figure 2 Comparison of Treg proportion and function in tumors between A-HCC and NA-HCC. **(A)** UMAP plot of re-clustered Tregs, with distinct clusters color-coded. **(B)** Bar plots showing proportions of Treg clusters in tumor and normal tissues across HCC subtypes. **(C)** Volcano plot of differentially expressed genes in Tregs between A-HCC and NA-HCC. Blue: downregulated; red: upregulated; gray: no significant change. **(D)** Dot plot of significantly enriched KEGG pathways from GSEA comparing tumor-derived Tregs. Positive NES (right dots) indicates enrichment in A-HCC; negative NES (left dots) indicates enrichment in NA-HCC. NES, normalized enrichment score.

Supplementary Figure 3 Prognostic value of A-HCC Treg-upregulated genes in HCC patients. **(A)** Forest plot for the TCGA Firehose Legacy cohort. **(B)** Forest plot for the TCGA PanCancer Atlas cohort. The hazard ratios (HR) were assessed using univariate Cox analyses. In each plot, each gene is shown as a box (HR) with horizontal lines (95% CI). The vertical line at HR = 1 indicates no effect; HR > 1 indicates worse prognosis.

Supplementary Figure 4 Evaluation of pro-inflammatory and anti-inflammatory functions of macrophages in A-HCC and NA-HCC. **(A)** Violin and box plots of pro-inflammatory and anti-inflammatory signature scores for Macro_c1 (left) and Macro_c2 (right) in normal (top) and tumor (bottom) tissues across HCC subtypes. p-values were calculated using two-tailed Wilcoxon rank-sum tests. **(B)** Volcano plot of differentially expressed genes in macrophages between A-HCC and NA-HCC. Blue: downregulated; red: upregulated; gray: no significant change.

Supplementary Figure 5 InferCNV analysis of copy number variations (CNV) in hepatocytes. T cells and myeloid cells were used as a normal reference. The plot shows inferred CNV scores in tumor hepatocytes relative to these reference populations.

Supplementary Figure 6 Differential in cell-cell communications and immunosuppressive ecosystem between A-HCC and NA-HCC. **(A)** Bar plot comparing the number and strength of inferred interaction between A-HCC and NA-HCC. **(B)** Heatmap of differential interaction strength among cell populations between the two subtypes.

Supplementary Figure 7 Validation of key findings using Harmony-integrated data. **(A)** UMAP plot of cell types in the Harmony-integrated dataset. **(B)** UMAP plot of sample distribution in the Harmony-integrated dataset. **(C)** Heatmap of marker gene expression across clusters. **(D)** Log_2_ fold change of immune cell proportions (Tumor vs. Normal) in A-HCC and NA-HCC. **(E)** Immunosuppression scores in Tregs across HCC subtypes and tissue types. **(F)** Pro-inflammatory and anti-inflammatory scores in macrophages across HCC subtypes and tissue types.

Supplementary Figure 8 Random subsampling analysis and pseudobulk analysis. **(A)** Random subsampling analysis of Treg proportion fold change (Tumor vs Normal) in NA-HCC. **(B)** Cell type proportions in pseudobulk analysis. Tumor vs. normal log_2_ fold change of immune cell types in A-HCC and NA-HCC. **(C)** Signature scores across HCC subtypes and tissue types in pseudobulk analysis. **(D)** CD8^+^ Te and NK/NKT cell glycolysis and TCA cycle scores across HCC subtypes and tissue types in pseudobulk analysis.

Supplementary Figure 9 Pseudobulk analysis of ligand-receptor gene expression in A-HCC and NA-HCC. **(A)** Gene expression of up-regulated genes in A-HCC in pseudobulk ananlysis. **(B)** Gene expression of up-regulated genes in NA-HCC in pseudobulk ananlysis.

## References

[1] Sung H Ferlay J Siegel RL Laversanne M Soerjomataram I Jemal A . Global cancer statistics 2020: Globocan estimates of incidence and mortality worldwide for 36 cancers in 185 countries. Ca-Cancer J Clin. (2021) 71:209–49. doi: 10.3322/caac.21660 33538338

[2] Ringelhan M Pfister D O'Connor T Pikarsky E Heikenwalder M . The immunology of hepatocellular carcinoma. Nat Immunol. (2018) 19:222–32. doi: 10.1038/s41590-018-0044-z 29379119

[3] Huang DQ Singal AG Kono Y Tan DJH El-Serag HB Loomba R . Changing global epidemiology of liver cancer from 2010 to 2019: Nash is the fastest growing cause of liver cancer. Cell Metab. (2022) 34:969–+. doi: 10.1016/j.cmet.2022.05.003 35793659 PMC9762323

[4] Zeng RW Ong CEY Ong EYH Chung CH Lim WH Xiao JL . Global prevalence, clinical characteristics, surveillance, treatment allocation, and outcomes of alcohol-associated hepatocellular carcinoma. Clin Gastroenterol H. (2024) 22:2394–402.e15. doi: 10.1016/j.cgh.2024.06.026 38987014

[5] Ganne-Carrié N Nahon P Chaffaut C N'Kontchou G Layese R Audureau E . Impact of cirrhosis aetiology on incidence and prognosis of hepatocellular carcinoma diagnosed during surveillance. Jhep Rep. (2021) 3:100285. doi: 10.1016/j.jhepr.2021.100285 34522876 PMC8424277

[6] Golabi P Rhea L Henry L Younossi ZM . Hepatocellular carcinoma and non-alcoholic fatty liver disease. Hepatol Int. (2019) 13:688–94. doi: 10.1007/s12072-019-09995-8 31701393

[7] deLemos A Patel M Gawrieh S Burney H Dakhoul L Miller E . Distinctive features and outcomes of hepatocellular carcinoma in patients with alcohol-related liver disease: A us multicenter study. Clin Transl Gastroenterol. (2020) 11:e00139. doi: 10.14309/ctg.0000000000000139 32352723 PMC7145044

[8] Kim SK Marusawa H Eso Y Nishikawa H Ueda Y Kita R . Clinical characteristics of non-B non-C hepatocellular carcinoma: A single-center retrospective study. Digestion. (2011) 84:43–9. doi: 10.1159/000333212 22156485

[9] Toshikuni N Tsutsumi M Arisawa T . Clinical differences between alcoholic liver disease and nonalcoholic fatty liver disease. World J Gastroenterol. (2014) 20:8393–406. doi: 10.3748/wjg.v20.i26.8393 25024597 PMC4093692

[10] Rorat M Hałoń A Jurek T . Histology of liver of the deceased due to harmful use of alcohol. Alcohol Alcohol. (2020) 55:518–23. doi: 10.1093/alcalc/agaa059 32626893

[11] Takahashi Y Fukusato T . Histopathology of nonalcoholic fatty liver disease/nonalcoholic steatohepatitis. World J Gastroenterol. (2014) 20:15539–48. doi: 10.3748/wjg.v20.i42.15539 25400438 PMC4229519

[12] Brunt EM . Alcoholic and nonalcoholic steatohepatitis. Clin Liver Dis. (2002) 6:399–420. doi: 10.1016/s1089-3261(02)00002-8 12122863

[13] Yip WW Burt AD . Alcoholic liver disease. Semin Diagn Pathol. (2006) 23:149–60. doi: 10.1053/j.semdp.2006.11.002 17355088

[14] Pinto HC Baptista A Camilo ME Valente A Saragoça A de Moura MC . Nonalcoholic steatohepatitis. Clinicopathological comparison with alcoholic hepatitis in ambulatory and hospitalized patients. Dig Dis Sci. (1996) 41:172–9. doi: 10.1007/bf02208601 8565753

[15] Haga Y Kanda T Sasaki R Nakamura M Nakamoto S Yokosuka O . Nonalcoholic fatty liver disease and hepatic cirrhosis: Comparison with viral hepatitis-associated steatosis. World J Gastroenterol. (2015) 21:12989–95. doi: 10.3748/wjg.v21.i46.12989 26675364 PMC4674717

[16] Basaranoglu M Basaranoglu G . Pathophysiology of insulin resistance and steatosis in patients with chronic viral hepatitis. World J Gastroenterol. (2011) 17:4055–62. doi: 10.3748/wjg.v17.i36.4055 22039318 PMC3203355

[17] Prieto J Melero I Sangro B . Immunological landscape and immunotherapy of hepatocellular carcinoma. Nat Rev Gastro Hepat. (2015) 12:681–700. doi: 10.1038/nrgastro.2015.173 26484443

[18] Zheng CH Zheng LT Yoo JK Guo HH Zhang YY Guo XY . Landscape of infiltrating T cells in liver cancer revealed by single-cell sequencing. Cell. (2017) 169:1342–+. doi: 10.1016/j.cell.2017.05.035 28622514

[19] Zhang Q He Y Luo N Patel SJ Han Y Gao R . Landscape and dynamics of single immune cells in hepatocellular carcinoma. Cell. (2019) 179:829–845.e20. doi: 10.1016/j.cell.2019.10.003 31675496

[20] Zhang QY Tsui YM Zhang VX Lu AJ Lee JMF Lee EV . Reciprocal interactions between Malignant cells and macrophages enhance cancer stemness and M2 polarization in HBV-associated hepatocellular carcinoma. Theranostics. (2024) 14:892–910. doi: 10.7150/thno.87962 38169544 PMC10758064

[21] Zheng B Wang DF Qiu XY Luo GJ Wu T Yang S . Trajectory and functional analysis of PD-1^high^ CD4^+^CD8^+^ T cells in hepatocellular carcinoma by single-cell cytometry and transcriptome sequencing. Adv Sci. (2020) 7:2000224. doi: 10.1002/advs.202000224 32670760 PMC7341083

[22] Ho DWH Tsui YM Chan LK Sze KMF Zhang X Cheu JWS . Single-cell RNA sequencing shows the immunosuppressive landscape and tumor heterogeneity of HBV-associated hepatocellular carcinoma. Nat Commun. (2021) 12:3684. doi: 10.1038/s41467-021-24010-1 34140495 PMC8211687

[23] Tang WW Sun GS Ji GW Feng TT Zhang Q Cao HS . Single-cell RNA-sequencing atlas reveals an FABP1-dependent immunosuppressive environment in hepatocellular carcinoma. J Immunother Cancer. (2023) 11:e007030. doi: 10.1136/jitc-2023-007030 38007237 PMC10679975

[24] Fu Y Maccioni L Wang XW Greten TF Gao B . Alcohol-associated liver cancer. Hepatology. (2024) 80:1462–79. doi: 10.1097/Hep.0000000000000890 38607725

[25] Cao L Wu D Qin L Tan D Fan Q Jia X . Single-cell RNA transcriptome profiling of liver cells of short-term alcoholic liver injury in mice. Int J Mol Sci. (2023) 24:1462–79. doi: 10.3390/ijms24054344 36901774 PMC10002329

[26] Chen SF Zhou YQ Chen YR Gu J . Fastp: An ultra-fast all-in-one fastq preprocessor. Bioinformatics. (2018) 34:884–90. doi: 10.1093/bioinformatics/bty560 30423086 PMC6129281

[27] Butler A Hoffman P Smibert P Papalexi E Satija R . Integrating single-cell transcriptomic data across different conditions, technologies, and species. Nat Biotechnol. (2018) 36:411–20. doi: 10.1038/nbt.4096 29608179 PMC6700744

[28] McGinnis CS Murrow LM Gartner ZJ . DoubletFinder: Doublet detection in single-cell RNA sequencing data using artificial nearest neighbors. Cell Syst. (2019) 8:329–337.e4. doi: 10.1016/j.cels.2019.03.003 30954475 PMC6853612

[29] Lu YM Yang AQ Quan C Pan YW Zhang HY Li YF . A single-cell atlas of the multicellular ecosystem of primary and metastatic hepatocellular carcinoma. Nat Commun. (2022) 13:4594. doi: 10.1038/s41467-022-32283-3 35933472 PMC9357016

[30] Korsunsky I Millard N Fan J Slowikowski K Zhang F Wei K . Fast, sensitive and accurate integration of single-cell data with Harmony. Nat Methods. (2019) 16:1289–+. doi: 10.1038/s41592-019-0619-0 31740819 PMC6884693

[31] Aran D Looney AP Liu LQ Wu E Fong V Hsu A . Reference-based analysis of lung single-cell sequencing reveals a transitional profibrotic macrophage. Nat Immunol. (2019) 20:163–+. doi: 10.1038/s41590-018-0276-y 30643263 PMC6340744

[32] Braun DA Street K Burke KP Cookmeyer DL Denize T Pedersen CB . Progressive immune dysfunction with advancing disease stage in renal cell carcinoma. Cancer Cell. (2021) 39:632–+. doi: 10.1016/j.ccell.2021.02.013 33711273 PMC8138872

[33] Sun YF Wu L Zhong Y Zhou KQ Hou Y Wang ZF . Single-cell landscape of the ecosystem in early-relapse hepatocellular carcinoma. Cell. (2021) 184:404–+. doi: 10.1016/j.cell.2020.11.041 33357445

[34] Kim DS Ryu JW Son MY Oh JH Chung KS Lee S . A liver-specific gene expression panel predicts the differentiation status of hepatocyte models. Hepatology. (2017) 66:1662–74. doi: 10.1002/hep.29324 28640507 PMC5698781

[35] Cheng Y-C Zhang Y Tripathi S Harshavardhan BV Jolly MK Schiebinger G . Reconstruction of single-cell lineage trajectories and identification of diversity in fates during the epithelial-to-mesenchymal transition. Proc Natl Acad Sci. (2024) 121:e2406842121. doi: 10.1073/pnas.2406842121 39093947 PMC11317558

[36] Lim E Wu D Pal B Bouras T Asselin-Labat ML Vaillant F . Transcriptome analyses of mouse and human mammary cell subpopulations reveal multiple conserved genes and pathways. Breast Cancer Res. (2010) 12:R21. doi: 10.1186/bcr2560 20346151 PMC2879567

[37] Infercnv of the Trinity Ctat Project. Available online at: https://github.com/broadinstitute/inferCNV (Accessed August 21, 2024).

[38] Jin S Guerrero-Juarez CF Zhang L Chang I Ramos R Kuan C-H . Inference and analysis of cell-cell communication using CellChat. Nat Commun. (2021) 12:1088. doi: 10.1038/s41467-021-21246-9 33597522 PMC7889871

[39] Ichiyama K Long J Kobayashi Y Horita Y Kinoshita T Nakamura Y . Transcription factor IKZF1 associates with FOXP3 to repress gene expression in Treg cells and limit autoimmunity and anti-tumor immunity. Immunity. (2024) 57:2043–2060.e10. doi: 10.1016/j.immuni.2024.07.010 39111316

[40] Zhang Q Wang HR Mao CY Sun M Dominah G Chen LY . Fatty acid oxidation contributes to IL-1β secretion in M2 macrophages and promotes macrophage-mediated tumor cell migration. Mol Immunol. (2018) 94:27–35. doi: 10.1016/j.molimm.2017.12.011 29248877 PMC5801116

[41] Lee JW Stone ML Porrett PM Thomas SK Komar CA Li JH . Hepatocytes direct the formation of a pro-metastatic niche in the liver. Nature. (2019) 567:249–+. doi: 10.1038/s41586-019-1004-y 30842658 PMC6430113

[42] Condeelis J Pollard JW . Macrophages: Obligate partners for tumor cell migration, invasion, and metastasis. Cell. (2006) 124:263–6. doi: 10.1016/j.cell.2006.01.007 16439202

[43] Noy R Pollard JW . Tumor-associated macrophages: From mechanisms to therapy. Immunity. (2014) 41:49–61. doi: 10.1016/j.immuni.2014.06.010 25035953 PMC4137410

[44] Bai Y Chen DP Cheng CL Li ZM Chi H Zhang YL . Immunosuppressive landscape in hepatocellular carcinoma revealed by single-cell sequencing. Front Immunol. (2022) 13:950536. doi: 10.3389/fimmu.2022.950536 35967424 PMC9365996

[45] Zimmerman KD Espeland MA Langefeld CD . A practical solution to pseudoreplication bias in single-cell studies. Nat Commun. (2021) 12:21038–1. doi: 10.1038/s41467-021-21038-1 33531494 PMC7854630

[46] Squair JW Gautier M Kathe C Anderson MA James ND Hutson TH . Confronting false discoveries in single-cell differential expression. Nat Commun. (2021) 12:25960–2. doi: 10.1038/s41467-021-25960-2 34584091 PMC8479118

[47] Yan GX Wang XF Sun C Zheng XD Wei HM Tian ZG . Chronic alcohol consumption promotes diethylnitrosamine-induced hepatocarcinogenesis via immune disturbances. Sci Rep-Uk. (2017) 7:2567. doi: 10.1038/s41598-017-02887-7 28566719 PMC5451469

[48] Wang QF Xia AN Luo CX Luo JH Zhao M Tang HR . Alcohol consumption affects macrophage polarization and promotes the Malignant behavior of gastric cancer cells by regulating the miR-29c-3p/Col1a1 signaling pathway. Cytotechnology. (2025) 77:194. doi: 10.1007/s10616-025-00862-z 41230538 PMC12602792

[49] Ceballos MP Rigalli JP Ceré LI Semeniuk M Catania VA Ruiz ML . ABC transporters: Regulation and association with multidrug resistance in hepatocellular carcinoma and colorectal carcinoma. Curr Med Chem. (2019) 26:1224–50. doi: 10.2174/0929867325666180105103637 29303075

[50] Hu B Zou T Qin W Shen X Su Y Li J . Inhibition of Egfr overcomes acquired lenvatinib resistance driven by Stat3-Abcb1 signaling in hepatocellular carcinoma. Cancer Res. (2022) 82:3845–57. doi: 10.1158/0008-5472.Can-21-4140 36066408 PMC9574378

[51] Hall Z Chiarugi D Charidemou E Leslie J Scott E Pellegrinet L . Lipid remodeling in hepatocyte proliferation and hepatocellular carcinoma. Hepatology. (2021) 73:1028–44. doi: 10.1002/hep.31391 32460431

[52] Gilglioni EH Li A St-Pierre-Wijckmans W Shen TK Pérez-Chávez I Hovhannisyan G . Ptprk regulates glycolysis and de novo lipogenesis to promote hepatocyte metabolic reprogramming in obesity. Nat Commun. (2024) 15:9522. doi: 10.1038/s41467-024-53733-0 39496584 PMC11535053

[53] Li H Wu K Tao KX Chen LB Zheng QC Lu XM . Tim-3/Galectin-9 signaling pathway mediates T-cell dysfunction and predicts poor prognosis in patients with hepatitis B virus-associated hepatocellular carcinoma. Hepatology. (2012) 56:1342–51. doi: 10.1002/hep.25777 22505239

[54] Yang RY Sun LL Li CF Wang YH Yao J Li H . Galectin-9 interacts with Pd-1 and Tim-3 to regulate T cell death and is a target for cancer immunotherapy. Nat Commun. (2021) 12:832. doi: 10.1038/s41467-021-21099-2 33547304 PMC7864927

[55] Hedrich V Breitenecker K Djerlek L Ortmayr G Mikulits W . Intrinsic and extrinsic control of hepatocellular carcinoma by Tam receptors. Cancers. (2021) 13:5448. doi: 10.3390/cancers13215448 34771611 PMC8582520

[56] Chen S Huang CS Li K Cheng MS Zhang CH Xiong JQ . Tumor-initiating cells escape tumor immunity via Ccl8 from tumor-associated macrophages in mice. J Clin Invest. (2025) 135:e180893. doi: 10.1172/JCI180893 39774471 PMC11870738

[57] Wang JY Zhao XQ Wan YSY . Intricacies of Tgf-β signaling in Treg and Th17 cell biology. Cell Mol Immunol. (2023) 20:1002–22. doi: 10.1038/s41423-023-01036-7 37217798 PMC10468540

[58] Zong Y Deng KH Chong WP . Regulation of Treg cells by cytokine signaling and co-stimulatory molecules. Front Immunol. (2024) 15. doi: 10.3389/fimmu.2024.1387975 38807592 PMC11131382

[59] Yu Y Li Y Zhou L Cheng X Gong Z . Hepatic stellate cells promote hepatocellular carcinoma development by regulating histone lactylation: Novel insights from single-cell Rna sequencing and spatial transcriptomics analyses. Cancer Lett. (2024) 604:217243. doi: 10.1016/j.canlet.2024.217243 39260669

[60] Wu L Yan J Bai Y Chen F Zou X Xu J . An invasive zone in human liver cancer identified by Stereo-Seq promotes hepatocyte-tumor cell crosstalk, local immunosuppression and tumor progression. Cell Res. (2023) 33:585–603. doi: 10.1038/s41422-023-00831-1 37337030 PMC10397313

