# Supplementary material for: Single-cell transcriptomic profile unveils distinct immune ecosystems in alcohol- and nonalcohol-associated hepatocellular carcinoma
Source: Front Immunol. 2026 Aug 18;17:1882068. doi: 10.3389/fimmu.2026.1882068 (PMC13529772; doi:10.3389/fimmu.2026.1882068)
Supplement: Supplementary file 1 [file Table1.docx]

**Table S1**. Clinical and pathological data of the two A-HCC patients included in scRNA-seq analysis.

| **Sample** | **Patient 1-Tumor** | **Patient 2-Tumor** |
| --- | --- | --- |
| **Gender** | Male | Male |
| **Age at diagnosis** | 52 | 65 |
| **Specimen type** | (Right) Partial hepatectomy specimen | (Right) Partial hepatectomy specimen |
| **Specimen size** | 6.1*4.9*3.3cm | 20*13*9cm |
| **Tumor size** | 1.6*1.2cm | ①10*8*7cm②1.5*1.2*1cm |
| **Histological type** | Moderately differentiated HCC | Moderately differentiated HCC |
| **Microvascular invasion** | M0 | M1(cancer thrombus in the portal vein at the hepatic hilum) |
| **Liver fibrosis classification** | Liver cirrhosis | Liver cirrhosis |
| **Clinical staging** | T1aN0M0, Ⅰa | T4N0M0, Ⅲb |
